# A Splice Mutation in the *PHKG1* Gene Causes High Glycogen Content and Low Meat Quality in Pig Skeletal Muscle

**DOI:** 10.1371/journal.pgen.1004710

**Published:** 2014-10-23

**Authors:** Junwu Ma, Jie Yang, Lisheng Zhou, Jun Ren, Xianxian Liu, Hui Zhang, Bin Yang, Zhiyan Zhang, Huanban Ma, Xianhua Xie, Yuyun Xing, Yuanmei Guo, Lusheng Huang

**Affiliations:** Key Laboratory for Animal Biotechnology of Jiangxi Province and the Ministry of Agriculture of China, Jiangxi Agricultural University, Nanchang, P.R. China; University of Bern, Switzerland

## Abstract

Glycolytic potential (GP) in skeletal muscle is economically important in the pig industry because of its effect on pork processing yield. We have previously mapped a major quantitative trait loci (QTL) for GP on chromosome 3 in a White Duroc × Erhualian F_2_ intercross. We herein performed a systems genetic analysis to identify the causal variant underlying the phenotype QTL (pQTL). We first conducted genome-wide association analyses in the F_2_ intercross and an F_19_ Sutai pig population. The QTL was then refined to an 180-kb interval based on the 2-LOD drop method. We then performed expression QTL (eQTL) mapping using muscle transcriptome data from 497 F_2_ animals. Within the QTL interval, only one gene (*PHKG1*) has a cis-eQTL that was colocolizated with pQTL peaked at the same SNP. The *PHKG1* gene encodes a catalytic subunit of the phosphorylase kinase (PhK), which functions in the cascade activation of glycogen breakdown. Deep sequencing of *PHKG1* revealed a point mutation (C>A) in a splice acceptor site of intron 9, resulting in a 32-bp deletion in the open reading frame and generating a premature stop codon. The aberrant transcript induces nonsense-mediated decay, leading to lower protein level and weaker enzymatic activity in affected animals. The mutation causes an increase of 43% in GP and a decrease of>20% in water-holding capacity of pork. These effects were consistent across the F_2_ and Sutai populations, as well as Duroc × (Landrace × Yorkshire) hybrid pigs. The unfavorable allele exists predominantly in Duroc-derived pigs. The findings provide new insights into understanding risk factors affecting glucose metabolism, and would greatly contribute to the genetic improvement of meat quality in Duroc related pigs.

## Author Summary

Glycogen storage diseases (GSD) are a group of inherited disorders characterized by storage of excess glycogen, which are mainly caused by the abnormality of a particular enzyme essential for releasing glucose from glycogen. GSD-like conditions have been described in a wide variety of species. Pigs are a valuable model for the study of human GSD. Moreover, pigs affected by GSD usually produce inferior pork with a lower ultimate pH (so-called “acid meat”) and less processing yield due to post-mortem degradation of the excess glycogen. So far, only one causal variant, *PRKAG3* R225Q, has been identified for GSD in pigs. Here we reported a loss-of-function mutation in the *PHKG1* gene that causes the deficiency of the glycogen breakdown, consequently leading to GSD and acid meat in Duroc-sired pigs. Eliminating the undesirable mutation from the breeding stock by a diagnostic DNA test will greatly reduce the incidence of GSD and significantly improve pork quality and productivity in the pig.

## Introduction

In past decades, thousands of quantitative trait loci (QTLs) have been detected for economically important traits in livestock through genetic linkage studies [1]. The recent availability of livestock genome sequences and high-density SNP chips has allowed detection of significant association of nucleotide polymorphisms with complex traits, and effective identification of causal mutations for some monogenic traits [2]–[4]. Despite these progresses, uncovering the quantitative trait genes (QTGs) or nucleotides (QTNs) for complex traits remains a challenging task. Only a handful of QTNs have been convincingly identified in livestock [5]–[7].

More recently, several studies in human, mouse and *Drosophila* have shown that the integration of phenotypic traits, genetic and gene transcript data enables researchers to identify expression QTL (eQTL), untangle gene-based regulatory networks, infer relationship between gene expression levels and phenotypic traits, and thereby detect novel trait-causing genes [8]–[10]. Therefore, the integrative analysis would play a key role in acquiring the knowledge of mechanisms responsible for livestock complex traits. Indeed, some researchers have identified a number of promising candidate genes for muscle traits and blood lipid traits of pig by integrative analyses of GWAS (or linkage mapping), eQTL and trait-correlated expression [11]–[14].

Glycogen storage diseases (GSD) characterized by defects in glycogen metabolism and excess glycogen stored in liver and muscle are multifactorial disorders. Both human and animals suffer from GSD. Agricultural researchers often measure the glycogen content or glycolytic potential [GP  = 2× (glucose + glycogen + glucose-6-phosphate) + lactate] in skeletal muscle of farm animals at slaughter. However, instead of the GSD diagnostic indicator, the GP value is mainly used for the prediction of meat quality development during the conversion of muscle to meat, because GP is a determinant of multiple meat quality characteristics, such as pH, color, water holding capacity (drip loss), tenderness and processing yields [15], [16].

GSD or extreme GP phenotype can be caused by genetic variation of various enzymes or transporters, which are involved either directly in the synthesis or breakdown of glycogen or in the utilization of its catabolite, glucose-1-phosphate [17]. To date, only one responsible gene, *PRKAG3*, has been elaboratively evidenced to impact glycogen and GP levels in Hampshire and its related synthetic lines [18], [19]. Although more than 10 different QTLs for the trait have been reported (http://cn.animalgenome.org/cgi-bin/QTLdb/index), QTGs or QTNs underlying these QTLs remain unexplored.

We have previously identified a major QTL for GP and pH values at 42 cM on chromosome 3 in a large scale White Duroc × Erhualian F_2_ intercross [20]. To decipher the molecular basis of the GP QTL, we herein performed an integrative genetic analysis by utilizing extensive data sets of GP-related traits, high-density genotypes and gene expression profiling from three experimental populations. We show compelling evidence that a splice mutation in the *PHKG1* gene is the QTN underlying the major QTL effect on GP and its related traits.

## Results

### Genome-wide association studies on two experimental populations define the QTL to a ∼180-kb region

**Figure 1:**
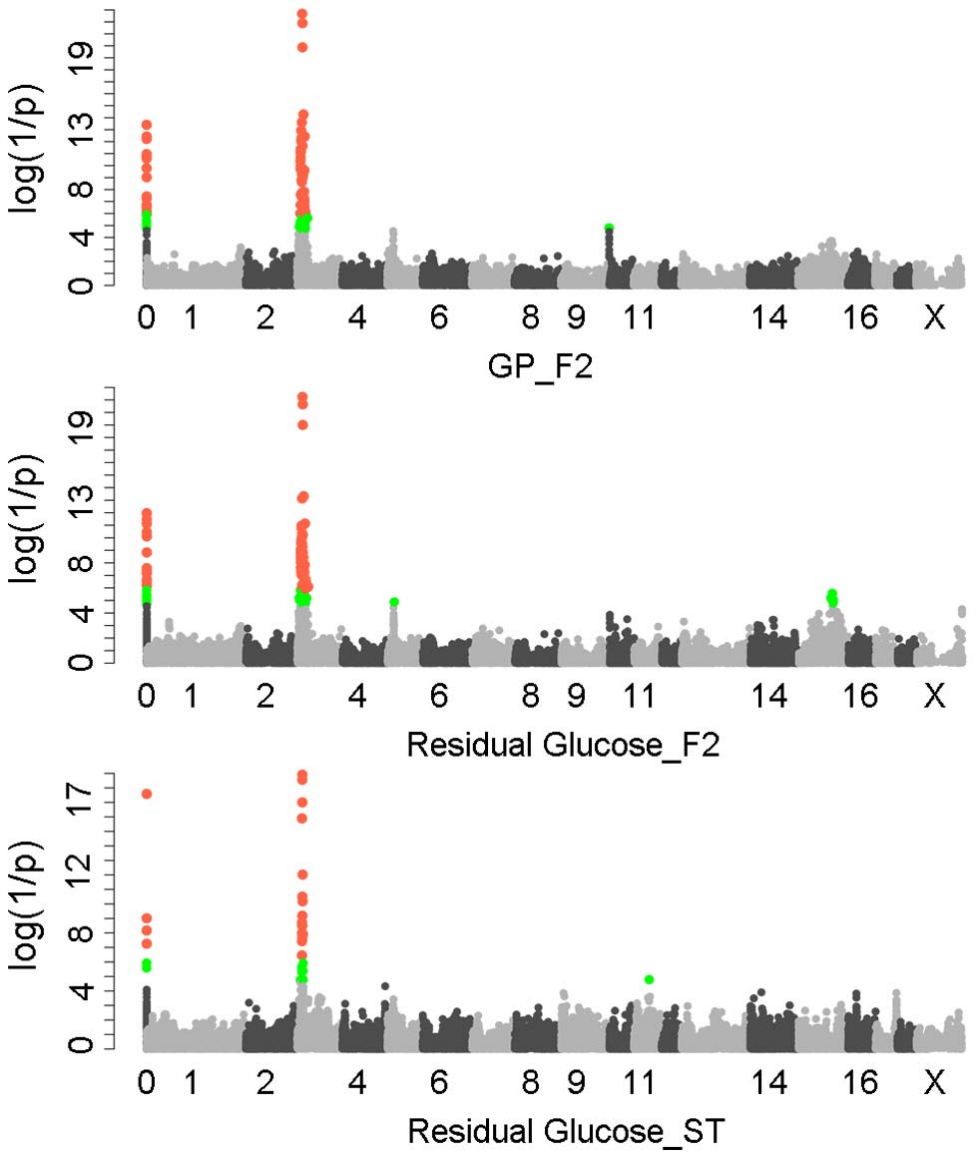
GWAS results for glycolytic potential and residual glycogen contents in longissimus muscle from the F_2_ and Sutai populations. In the Manhattan plots, log_10_(1/P) values of the qualified SNPs were plotted against their genomic positions. “Chromosome 0” harbors SNPs that have not yet been mapped to the *Sus Scrofa* 10.2 assembly. It is most likely that the significant SNPs on chromosome 0 are located on chromosome 3 and have strong linkage disequilibrium with the top SNPs ss131031160 or ss131565361. The red and green dots represent the SNPs that reached 5% genome-wide and suggestive Bonferroni-corrected significances, respectively. The most significant SNPs for residual glycogen are ss131031160 and ss131565361 on SSC3 in the F_2_ and Sutai (ST) pigs respectively. GP, glycolytic potential.

We first performed a genome-wide association study (GWAS) for GP and its components including residual glycogen & glucose (RG), glucose-6-phosphate (G-6-P) and lactate on the White Duroc × Erhualian F_2_ intercross, in which 877 phenotyped F_2_ individuals and its parents/grandparents were genotyped by using Illumina Porcine SNP 60K Beadchips [21]. The quality control filtering and data processing of the GWAS data are described in the Methods. Quantile-quantile plots with genome control λ_GC_ values are shown in Figure S1. We found no evidence of systematic inflation of association test results. Consistent with our previous QTL results [20], the GWAS results demonstrated that Sus Scrofa chromosome 3 (SSC3) contained the most genome-wide significant locus (*P* = 5.85×10^−22^) for both GP and RG with the top SNP ss131031160 (pSNP) at 17.09 Mb (Figure 1). However, this lead SNP was not associated with the glycolysis intermediate product G-6-P and end-product lactate (Figure S2). These results suggest that the underlying QTG likely influences the conversion between glycogen and glucose (or G-6-P) rather than the conversion between G-6-P to lactate. The pSNP ss131031160 explains 19.6% of the phenotypic variance in RG of the F_2_ population.

**Figure 2:**
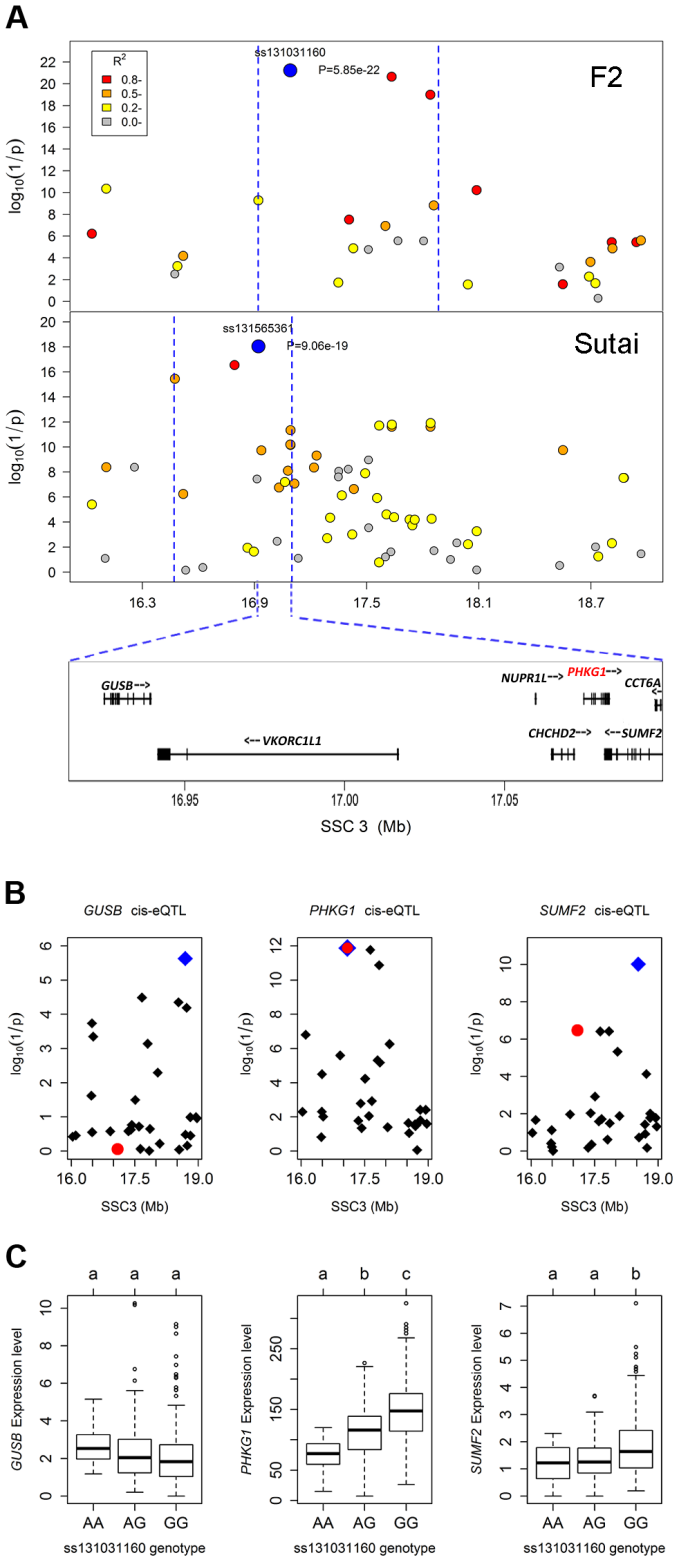
Prioritizing candidate genes by the colocalization between pQTL and eQTL. (A) Regional association plots with residual glycogen content in longissimus muscle from the F_2_ (top panel) and Sutai (bottom panel) populations. The top pSNPs ss131031160 (F_2_) and ss131565361 (Sutai) are highlighted by blue dots. Different levels of linkage disequilibrium (LD) between the top SNPs and surrounding SNPs are indicated by different colours. The QTL intervals indicated by blue dash lines were obtained by the 2-LOD drop method. Their overlapping region spans 180 kb and contains 7 annotated genes. (B) Manhattan plots for the genome-wide cis-eQTL analysis of three candidate genes *GUSB* (left), *PHKG1* (middle) and *SUMF2* (right) in the F_2_ population. The blue diamonds represent the top cis-eSNPs, while the red dot represents the top pSNP ss131031160 in the same population. (C) Box plots showing the differences in gene expression level determined by the digital gene expression (DGE) system among three genotypes of the ss131031160 SNP. Different letters above each box plot denote significant differences (*P*<0.05).

To validate and fine map the SSC3 QTL for RG, we conducted a second GWAS on 433 Sutai pigs, a Chinese synthetic line derived from a cross between Duroc and Taihu including Erhualian and Meishan after more than twenty-year selection. Besides 62163 SNPs on the Illumina Porcine SNP 60K Beadchip, 53 SNPs derived from DNA sequence comparison between Erhualian and Duroc (see Materials and Methods) within a 1-Mb region surrounding the top pSNP were genotyped for these Sutai pigs. As expected, we confirmed the strong association signal on SSC3 in the Sutai population. The pSNP ss131565361 (*P* = 9.06×10^−19^) at 16.92 Mb (Figure 1) explains 53.6% of the RG variance. This locus also significantly influenced ultimate pH (measured 24 hours after slaughter) and drip loss (Figure S2). Based on the LOD drop off 2, the empirical confidence intervals of the QTL in the F_2_ and Sutai populations was 920 kb (16.92–17.84 Mb) and 630 kb (16.47–17.10 Mb) respectively (Figure 2A). Therefore, the most likely QTL interval was their overlapping region of 180 kb (16.92–17.10 Mb). This interval contains 7 annotated genes: *GUSB*, *VKORC1L1*, *NUPR1L*, *CHCHD2*, *PHKG1*, *SUMF2* and *CCT6A* (Figure 2A).

### eQTL mapping supports *PHKG1* as the most likely responsible gene

Using the data of digital gene expression profiles (DGE) tested in longissimus muscle samples from 497 F_2_ animals, we identified genome-wide eQTL for the 7 annotated genes in the critical region. We operationally defined cis-eQTL as any association between a gene expression and SNPs within 2 Mb of the gene location. We found 3 cis-eQTL each for *PHKG1*, *SUMF2* and *GUSB* (Figure 2B). Intriguingly, the lead SNP for the cis-eQTL affecting *PHKG1* expression was identical to the top pSNP (ss131031160) for GP in the GWAS on the F_2_ population. The major allele (G) at this SNP was associated with lower residual glycogen and higher expression of *PHKG1* (Table 1 and Figure 2C). *PHKG1* encodes a catalytic subunit of the phosphorylase kinase (PhK), which can mediate glycogen breakdown. Therefore, the co-localization of the top SNP in both GWAS and eQTL mapping coupled with the biological function highlights *PHKG1* as the most likely QTG underlying the major locus on SSC3.

**Table 1:** The effect of the SSC3 QTL on glycolytic potential and its components in the White Duroc × Erhualian F_2_ intercross population^a^.

QTL genotype	Number	GP (µmol/g)	Residual G (µmol/g)	G-6-P (µmol/g)	Lactate (µmol/g)
QQ (AA)	45	186.00±31.45^a^	46.75±11.90^a^	0.18±0.38^a^	92.12±25.74^a^
Qq (AG)	344	140.88±26.42^b^	24.95±13.68^b^	0.18±0.45^a^	90.61±21.93^a^
qq (GG)	475	129.80±24.74^c^	20.99±12.25^c^	0.15±0.28^a^	87.53±21.75^a^
Additive effect		28.10	12.88		
Dominance effect		−17.02	−8.92		

a QTL genotypes correspond to the genotypes of the most significant SNP ss131031160 for GP, which are given in brackets. GP, glycolytic potential that is calculated by using the formulae: GP  =  2× (residual G + G-6-P) + lactate; Residual G, combination of glycogen and glucose in muscle; G-6-P, glucose-6-phosphate. Values with different superscripts in a column are significantly different from each other (*P*<0.05).

### Sequencing the *PHKG1* gene identifies a splice mutation as candidate QTN

**Figure 3:**
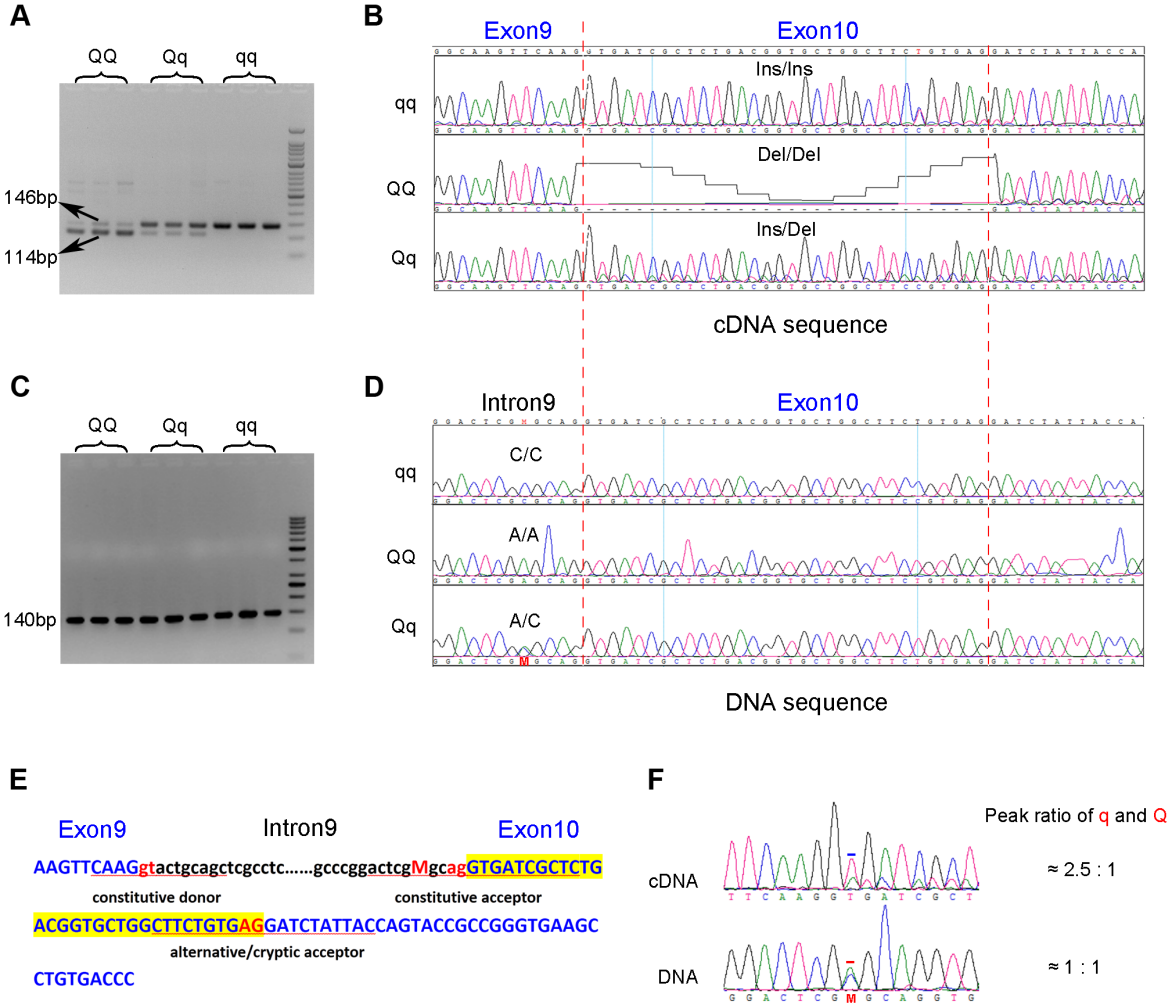
Identification of the g.8283C>A splice mutation in the *PHKG1* gene. (A) RT-PCR products of the *PHKG1* gene on a 2.5% agarose gel, amplified from total RNA from animals with different QTL genotypes (*QQ*, *Qq* and *qq*). Two DNA bands of 146 bp and 114 bp with different intensity were observed. (B) Sequence analysis of the RT-PCR products. A 32-bp deletion in exon 10 was detected in *QQ* and *Qq* animals. (C) PCR products of the *PHKG1* gene on a 2.5% agarose gel, amplified from genomic DNA from animals with different QTL genotypes (*QQ*, *Qq* and *qq*). (D) Sequence analysis of the PCR products. A point mutation (g. 8283C>A) was identified in *QQ* and *Qq* individuals. (E) *PHKG1* genomic sequence from exon 9 to exon 10. Intronic sequence is in black and lower case, and exonic sequence in blue and upper case. The normal splice-acceptor and -donor sequences (lower case) and the cryptic splice-donor sequences (upper case) are underlined with two red nucleotide letters in the center. The C>A mutation is denoted as “M” in red. (F) Sequence analysis of a heterozygote at the g.8283C>A mutation site. In genomic DNA from a heterozygote, the C and A peaks (the position is indicated by “M” and marked by the red bar) were of equal height in sequence of genomic DNA, as expected. Sequence of the cDNA product from the same heterozygote showed a major peak corresponding to the wild-type sequence and a small peak corresponding to the mutant sequence (the +2 position of exon 10 where the nucleotide *T* belong to the 32-bp deleted portion is marked by the blue bar).

To search for coding variants in the *PHKG1* gene, we isolated and sequenced *PHKG1* cDNA from muscle samples of 6 F_2_ individuals representing three genotypes at the pSNP ss131031160 (2 *GG*, 2 *GA* and 2 *AA* animals). In total, we detected 14 polymorphisms (Table S1). Of note, we found a 32-bp deletion/insertion (c.del/ins32; Figure 3A&B) polymorphism at exon10 that alters the open-reading frame and causes a premature stop codon, leading to a truncated and nonfunctional protein product.

To test if the 32-bp deletion was also present in the genomic DNA (gDNA), we sequenced the 140 bp genomic region encompassing the deletion using gDNA samples of the above-mentioned 6 F_2_ animals. We found only one single nucleotide substitution (C>A) in the region. This SNP (g.8283C>A) lies 5 bp upstream of the c.del/ins32 (Figure 3C&D), where the pyrimidine (C or T) is highly conserved among vertebrates, including chicken, mouse, dog, cow, sheep and human. The C→A nucleotide transvertion may weaken the strength of the pyrimidine-rich signal near the 3′ end of the *PHKG1* intron 9, which is known to correlate with splicing efficiency [22]. Using a web-available tool Alternative Splice Site Predictor (ASSP, http://wangcomputing.com/assp/index.html), we found that the splice site score (1.652) of the mutant sequence was much lower than the wild-type counterpart (3.659), and even lower than the default cutoff value (2.2) for acceptor sites set in the predictor. Therefore, the mutation appears to attenuate or inhibit the role of the constitutive acceptor splice site and this consequently evokes another cryptic acceptor at 32 bp downstream of the mutation, which results in a loss of 32 bp in mature mRNA during *PHKG1* transcription (Figure 3E).

To test if the 32-bp deletion in exon 10 was directly caused by the SNP g.8283C>A, the effect of the variant on splicing was assessed using a minigene splicing assay (Figure S3). Two minigene expression vectors carrying either the wild-type or the mutant *PHKG1* g. 8283C>A segment were constructed and transiently transfected into HeLa cells and 293T cells (Figure S3A). The minigene transcripts in these transfected cells were analyzed by RT-PCR, using specific primers complementary to exon 9 and exon 10 of the minigene. We found two amplicons of 78 bp and 62 bp from the mutant construct and only one amplicon of 94 bp from the wild-type construct (Figure S3B). Sanger sequencing analysis revealed that the two truncated amplicons corresponded to two aberrant splicings of the first 16 and 32 nucleotides (nt) at 5′ end of exon 10 (Figure S3C-E). The presence of novel aberrant splicing of 16-nt may be due to that the minigene constructs only contain partial *PHKG1* sequence or there is a special splicing factor in the transfected cells. Anyway, the result clearly demonstrates that the variant *PHKG1* g. 8283C>A is responsible for the aberrant splicing of 32-nt observed in vivo.

### The candidate QTN co-segregates with QTL genotypes of parental boars in the two experimental populations

To obtain further evidence for the causality of the candidate QTN (SNP g.8283C>A), we conducted the concordance test between the SNP genotypes and the QTL genotypes on 9 parental boars (6 F_1_ boars and 3 Sutai boars). The QTL genotypes of these boars were determined by the marker-assisted segregation analysis (see Materials and Methods). We found that the SNP genotypes were completely concordant with the QTL genotypes in these boars (Table S2). In contrast, the two top pSNPs ss131031160 and ss131565361 in the GWAS exhibited disconcordance with the QTL genotypes in the 9 parental sires. Concordantly, the association of the g.8283C>A SNP with RG phenotype was strongest among all SNPs genotyped in Sutai pigs, and was at the same significance level as that of the top GWAS SNP ss131031160 in the F_2_ population due to their complete linkage disequilibrium (Table S3).

In addition, we re-sequenced a 10-kb segment covering 1 kb upstream of the *PHKG1* gene to its 3′ end on all 9 sires (6 F_1_ and 3 Sutai sires) with deduced QTL status. A total of 142 variants were identified (Table S4). We found that 7 *Q* chromosomes of these boars shared the same haplotype while 11 *q* chromosomes corresponded to multiple divergent haplotypes (Figure S4). Surprisingly, a *q* haplotype from Sutai boars was nearly identical to the *Q* haplotypes except for three variants, of which only one (g.8283C>A) showed co-segregation with QTL genotypes across all the 9 sires (Figure S5). This strongly supports the causality of the g.8283C>A mutation.

Haplotype analysis showed that no other variants within the *PHKG1* region were in complete LD with the SNP g.8283C>A across all experimental populations. Based on that, we tried to determine whether other variants can also result in partial QTL effect. When the SNP g.8283C>A was included as a fixed effect in the model, the QTL effects on *PHGK1* expression and RG trait vanished in these populations (Figure S6A). Furthermore, we divided the Sutai pigs into three genotype groups (*AA*, *CA*, *CC*). No eQTL effect was detected within each group (Figure S6B). It thus suggests that even there is another regulatory SNPs, their effects are likely negligible.

### The QTN significantly affects *PHKG1* mRNA and protein levels

Aberrant mRNA, like the truncated *PHKG1* transcript caused by the g.8283C>A SNP, tend to be degraded by a known mechanism of nonsense-mediated mRNA decay (NMD) in the cell. To analyze whether the mutation interferes with *PHKG1* mRNA expression levels, qRT-PCR experiments were performed on total RNA isolated from 293T cells transiently transfected by the wild-type and mutant *PHKG1* minigenes. The mutant minigene produced nonsense mRNAs at 56% level of normal expression (Figure S7). The finding indicates that the *PHKG1*-32del mRNA bearing the premature termination codon was most likely degraded by NMD.

**Figure 4:**
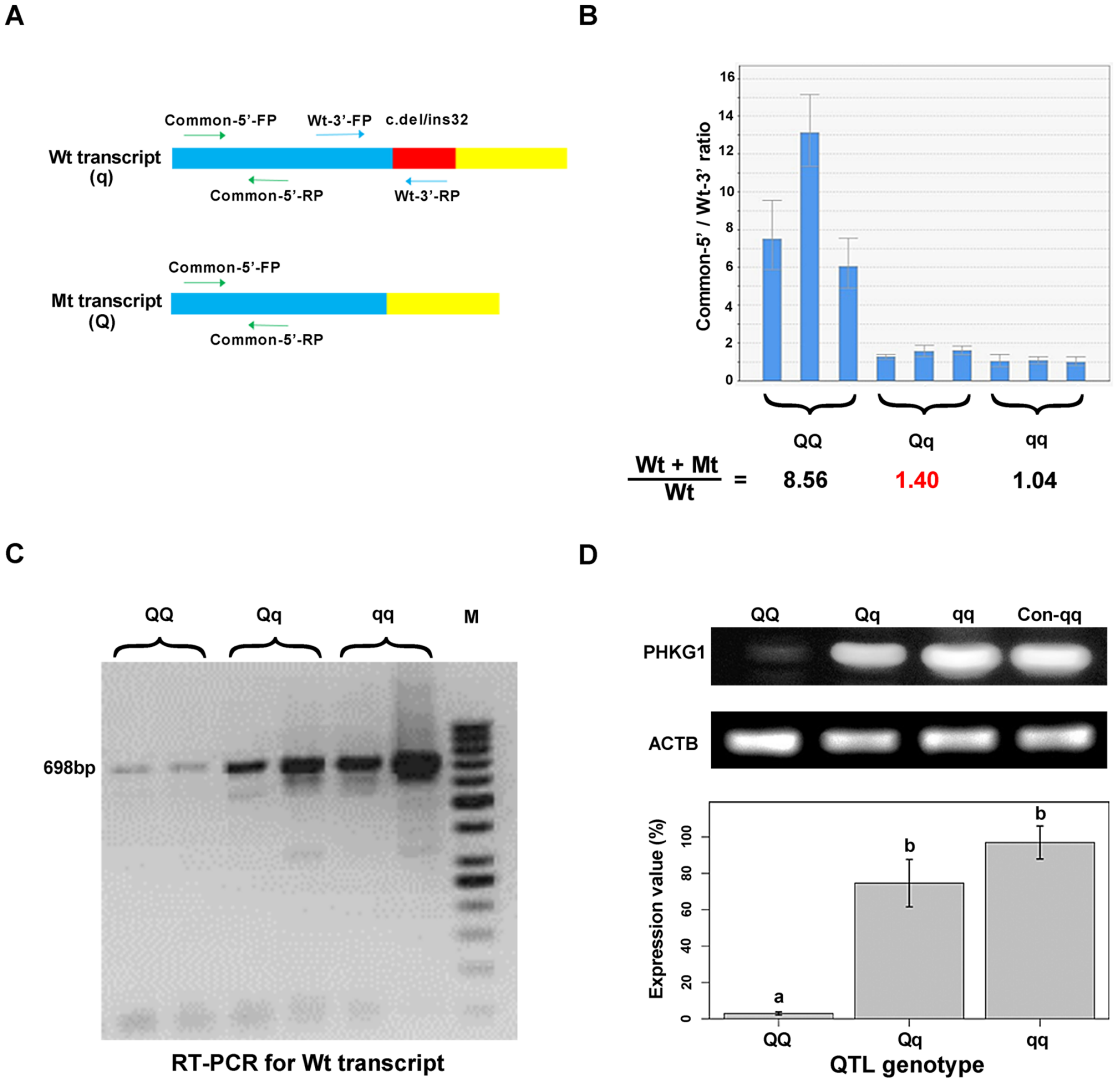
Variation in *PHKG1* expression at transcript and protein levels. (A) The schematic illustration of the match positions of RT-PCR primers. The primer set Common-5′-FP/RP matches the 5′-end of wild-type (Wt or q) and mutant (Mt or Q) transcripts of *PHKG1*. The primer set Wt-3′-FP/RP matches the 3′-end of Wt, whereas the Wt-3′- RP mismatches the 3′-end of Mt. (B) The ratio of Wt+Mt and Wt transcripts determined by qRT-PCR with the Common-5′- and Wt-3′-primer sets in 9 muscle samples (3 per QTL genotype). (C) Electrophoresis of RT-PCR products showing the presence and abundance of 698-bp cDNA (transcribed from the *PHKG1* Wt) in animals with three QTL genotypes. (D) Western blot analysis showing PHKG1 protein levels in animals with *QQ*, *Qq* and *qq* genotypes. The level of “Con-qq” served as a calibrator sample for all experiments. The expression level of β-Actin (ACTB) was used as a loading control. PHKG1 protein levels from six independent experiments were quantitated by the GeneTools analysis software. Histogram (lower panel) shows the amount of PHKG1 protein relative to the “Con-qq” control after normalization to that of ACTB.

In fact, electropherogram of RT-PCR products amplified from *PHKG1* mRNA in homozygous animals showed that the intensity of the mutant DNA band of 114 bp was much lower than the normal band of 146 bp (Figure 3A), implying NMD of the mutant *PHKG1* mRNA. Accordingly, the *PHKG1* cDNA sequence chromatograms illustrated that the fluorescence intensity of wild-type allele (Wt or q) were 2–3 fold stronger than that of the mutant allele (Mt or Q) in animals heterozygous for the g.8283C>A SNP (Figure 3F). To more accurately estimate the difference in abundance between Wt and Mt transcripts, we designed two pairs of primers: one (Common-5′-FP/RP) for amplification of both transcripts, and the other (Wt-3′-FP/RP) for the specific amplification of Wt transcript (Figure 4A). After performing qRT-PCR with the two primer sets, we quantified the levels of total mRNA transcripts relative to wild-type transcripts. In heterozygotes, the ratio of (Mt+Wt) to Wt transcripts was 1.4∶1 (Figure 4B), suggesting that the 60% of Mt transcripts were degraded by NMD.

Interestingly, we found that mutant homozygotes (*AA* or *QQ*) had *PHKG1* Wt, as RT-PCRs using the primers specific for the Wt generated amplicons corresponding to Wt in these mutant homozygotes (Figure 4C). We further showed that, only about one-eighth of *PHKG1* transcripts was Wt (Figure 4B). The result suggests that the g.8283C>A mutation could strongly decrease but not completely abrogate the original splicing form.

Consistently, the *PHKG1* Wt transcript level is highest in muscle samples from *CC* animals, followed by *AC* and *AA* individuals (Figure 4C). Similar tendency was observed for the PHKG1 Wt protein level in the three genotypes by Western-blot analysis (Figure 4D). Thus, it is evident that the g.8283C>A mutation affects the expression of *PHKG1* at both transcription and translation levels.

### *PHKG1* mRNA level is significantly correlated with the RG phenotype

**Figure 5:**
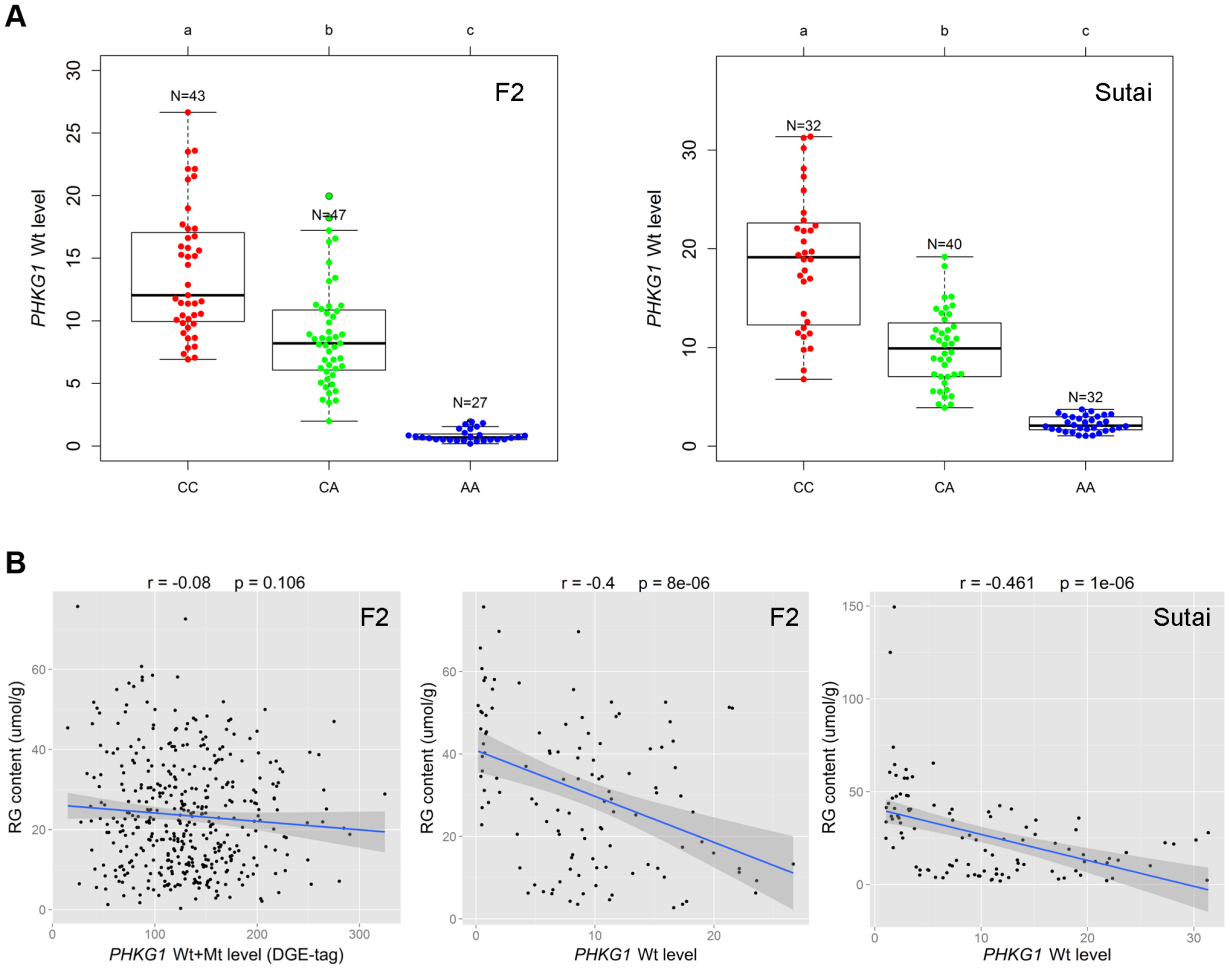
Relationship between the *PHKG1* transcript level and residual glycogen content. (A) Plots showing the association of the *PHKG1* g.8283 A>C genotype with the *PHKG1* wild-type transcript (Wt) levels that were measured by quantitative RT-PCR using muscle samples from 117 F_2_ pigs (left panel) and 104 Sutai pigs (right panel). Different letters above each box plot denote significant differences (*P*<0.05). (B) The strength of associations between residual glycogen (RG) content and the total transcript level of *PHKG1* from the DGE data of 412 F_2_ animals (r = −0.08, *P* = 0.106; left panel), and between RG and the *PHKG1* Wt level tested in 117 F_2_ animals (r = −0.4, *P* = 8×10^−6^; middle panel) and 104 Sutai pigs (r = −0.461, *P* = 1×10^−6^; right panel).

*PHKG1* is a critical enzyme in the glycogen metabolism. We expected that the *PHKG1* mRNA expression level would be significantly associated with the RG phenotype. However, we did not observe such strong correlation (r = −0.08, *P* = 0.106; Figure 5B) using the DGE data in the F_2_ population. We then made a close examination on the DGE data. We found that the DGE tag for *PHKG1* was located in a region upstream of the 32-bp deletion, which therefore can not distinguish Wt from Mt transcripts. To correct for the biased effect of the abnormal Mt transcripts, we further used qRT-PCR to measure the Wt level on 117 F_2_ individuals and 104 Sutai pigs, of which the number of *CC*, *CA* and *AA* animals at the g.8283C>A locus are nearly identical (Figure 5A). We then examined the correlation between the qPCR-data and the RG phenotypes. As a result, the *PHKG1* Wt (functional) transcript level was significantly correlated with RG phenotype (r≤−0.4, *P*<10^−5^) in the two populations (Figure 5B). Such strong correlation were not detected for any other cis-regulated genes within the critical region of the SSC3 QTL (Figure S8), strengthening the causal relationship between *PHKG1* and RG.

### The *PHKG1* g.8283C>A mutation significantly alters the enzymatic activity of the phosphorylase kinase

**Figure 6:**
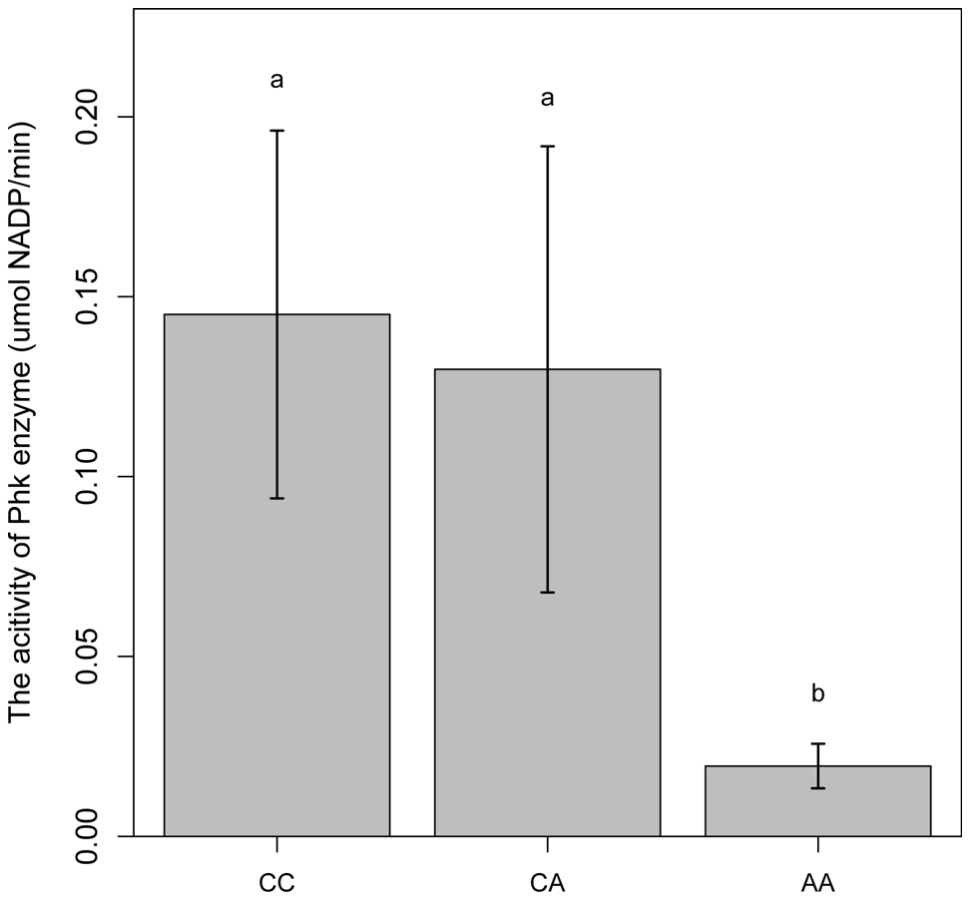
Comparison of PhK enzyme activities in longissimus muscle of animals with different *PHKG1* g.8283 A>C genotypes. Bars with different letters are significantly different. Data are means ±1 SEM (n = 6 per genotype).

PHKG1 is a catalytic subunit of the phosphorylase kinase (PhK). The g.8283C>A splice mutation in the *PHKG1* gene thus likely influence the enzymatic activity of PhK and glycogen phosphorylase. To verify the assumption, we tested the enzymatic activity of PhK in 18 muscle samples―6 per genotype group (*CC*, *AC*, and *AA*). As expected, the *AA* group showed>6-fold reduction in enzyme activity as compared to the *CC* and *AC* groups (Figure 6). It is reasonable to speculate that reduced PhK enzyme's activity would lead to slow breakdown of glycogen in muscle, which in turn cause excess accumulation of glycogen in the tissue. This is consistent with the SSC3 QTL effect on GP or RG trait.

### The *PHKG1* g.8283C>A mutation is significantly associated with GP-related meat quality traits in multiple Duroc-derived populations

Considering the inter-relationship of GP with other meat quality attributes, we evaluated the effects of the *PHKG1* g.8283C>A mutation on all meat quality traits measured in the 864 F_2_ individuals from the White Duroc × Erhualian intercross, 431 Sutai pigs and 140 three-way hybrid DLY [Duroc × (Landrace × Yorkshire)] pigs. In the F_2_ population, there was a tendency towards lower ultimate pH and higher drip loss in *AA* pigs with higher GP compared to *CA* and *CC* individuals (the mean values of pH 24h in *AA*, *CA* and *CC* groups were 5.70, 5.72 and 5.77, respectively; for drip loss: 1.01%, 0.85% and 0.92%, respectively), although the mean differences did not reach statistical significance, partially due to a low frequency (5%) of the *AA* genotype in the population.

In Sutai pigs, each genotype group (*AA*, *AC* or *CC*) at the *PHKG1* g.8283C>A site has more than 100 individuals. Significant effects of the causal mutation were observed on almost all meat quality traits in this population (Tables 2 and S5). Especially, the RG level in the longissimus muscle (LM) of *AA* animals was 4 times higher (*P* = 9.03×10^−54^) than those in *CC* animals. Coincidently, drip loss, rate of pH decline and Minolta a* and b* were higher (*P*<0.01) in *AA* pigs than *CC* pigs. *AA* pigs also had lower (*P*<0.001) intramuscular fat content (IMF) and marbling score compared to *CC* pigs. In the DLY hybrid commercial population, all 140 individuals were genotyped for the *PHKG1* g.8283C>A mutation and 53 surrounding SNPs using the OpenArray platform (see Materials and Methods). Again, we observed the significant effects of the g.8283C>A mutation on RG, pH and drip loss (Table 2). Moreover, this mutation showed stronger associations with these traits than any other surrounding SNPs (Figure S9). These results indicate that the *A* allele has unfavorable effects on these meat quality traits.

**Table 2:** Effect of *PHKG1* g.8283C>A on meat quality traits of longissimus muscle in Chinese synthetic Sutai pigs and Western commercial Duroc × (Landrace × Yorkshire) hybrid pigs.

	Mean ± standard error b			
Traits a	AA (n)	AC (n)	CC (n)	*P* value
**Sutai pigs**				
Residual G of LM, µmol/g	44.60±19.35^a^ (110)	15.91±13.34^b^ (204)	11.74±10.39^c^ (105)	9.03E-54
pH 24h of LM	5.55±0.13^a^ (96)	5.59±0.19^a^ (153)	5.67±0.23^b^ (91)	2.42E-07
24-h drip loss of LM, %	2.89±1.63^a^ (122)	2.56±2.12^a^ (203)	1.99±1.76^b^ (104)	4.42E-07
IMF of LM, %	1.21±0.56^a^ (119)	1.61±0.72^b^ (204)	1.77±0.67^b^ (105)	4.96E-04
Subjective marbling score of LM, 1-10	1.97±0.53^a^ (122)	2.27±0.53^b^ (203)	2.40±0.53^b^ (104)	2.30E-04
**DLY pigs**				
Residual G of LM, µmol/g	20.84±11.50 (9)	16.54±12.44 (46)	11.04±8.36 (85)	0.006
pH 36h of LM	5.49±0.31 (7)	5.41±0.17 (42)	5.43±0.23 (61)	0.011
24-h drip loss of LM, %	4.34±2.23 (7)	4.05±2.06 (42)	3.42±2.00 (63)	0.002
IMF of LM, %	1.60±0.30 (9)	1.90±0.70 (46)	1.60±0.50 (85)	0.827
Subjective marbling score of LM, 1-10	2.33±0.56 (9)	2.71±0.61 (46)	2.82±0.71 (85)	0.088

a LM, longissimus muscle; Residual G, glycogen + glucose; pH 24h and pH 36h, pH value measured at postmortem 24h and 36h respectively; IMF, Intramuscular fat content; DLY, Duroc × (Landrace × Yorkshire).

b Values with different superscripts in a row are significantly different from each other (*P*<0.05).

To reveal the allele frequency of the *PHKG1* g.8283C>A mutation in diverse pig breeds, we genotyped this mutation in a broad panel of 629 animals representing 5 European commercial breeds, 2 Chinese domestic breeds, and wild boars from China and Europe. The *A* allele causing excess glycogen content occurred at high frequency (0.70) in White Duroc and at medium frequency (0.32) in Red Duroc, but it was nearly absent in other domestic and wild breeds (<0.03; Table 3). The *PHKG1* mutant allele was absent in wild boars, suggesting that the mutation likely happened after domestication. However, due to limit number of wild boars examined in this study, we cannot rule out the possibility that it is a standing variation.

**Table 3:** Allele frequency distribution of *PHKG1* g.8283C>A in Western and Chinese pigs.

Breeds	N	Frequency of the mutant allele (A)
**Western pigs**		
White Duroc	10	0.70
Duroc	325	0.32
Yorkshire (Large White)	60	0.03
Pietrian	107	0.01
Landrace	48	0.00
Wild boar	12	0.00
**Chinese pigs**		
Laiwu	24	0.00
Erhualian	24	0.00
Wild boar	19	0.00

## Discussion

To date, only a handful of QTG and QTN have been identified for complex traits in farm animals. To the best of our knowledge, this is the first study that has used a system genetics approach including GWAS, eQTL mapping and causality modeling to identify QTG and QTN for complex traits in pigs.

Our study supports the *PHKG1* gene as a QTG for GP-related traits based on the following findings: (1) GWAS on the F_2_ and Sutai populations enable us to define the major QTL for GP within an interval of 180-kb on SSC3, which contains only 7 genes including *PHKG1*. (2) Of three positional candidate genes with cis-eQTLs signals, only *PHKG1* has the cis-eQTL peak SNP that is identical to the pQTL peak SNP. (3) PHKG1 is a catalytic subunit of the phosphorylase kinase (PhK), which is critical to glycogen degradation. (4) The wild-type transcript level of *PHKG1* was significantly correlated to glycogen content in muscle.

Furthermore, our study shows the following evidences for the *PHKG1* g.8283C>A mutation as QTN underlying the SSC3 QTL effect: (1) The mutation caused abnormal splicing of *PHKG1* mRNA, resulting in a shift in the reading frame with a premature stop codon. (2) The aberrant mRNA transcripts were diminished by NMD, which reduced the PHKG1 protein level and led to PhK enzyme deficiency. (3) The mutation shows the complete concordance between their genotypes and the deduced QTN genotypes across all parental boars. No other variants in the *PHKG1* gene exhibited such concordance. (4) This mutation has a consistent and significant effect on GP-related traits across all tested populations.

Here, we illustrate that the QTN (g.8283C>A) is a splice site mutation affecting both transcription and translation levels of the *PHKG1* gene, which has at least three implications. One is that cis-eQTL mapping for annotated genes residing the QTL region can not only contribute to identification of regulatory QTNs, but also benefit characterization of protein-altering QTNs including splice and nonsense mutations that affect transcript levels by NMD, just like our identified QTN. Another implication is that if a gene is subjected to alternative splicing, it is necessary to determine the levels of its different transcripts (instead of only the total amount of these transcripts) by deep RNA sequencing and then examine their individual relationship with phenotypic traits. The third one is that we could identify a cis QTL for PHKG1 protein level that overlaps with the mapped cis-eQTL and pQTL on SSC3 if a big data set on protein abundance was available, and find a strong relationship between protein level and GP-related traits. This information could add a new dimension for the search of QTG. Several studies have recently demonstrated the feasibility of high-throughput proteome quantification and revealed the variation and genetic control of protein abundance in humans and mice [23], [24]. Hopefully, whole proteome analysis will be more efficient and routinely added to integrative genomic analysis to accelerate the identification of QTG and QTN for complex traits in livestock.

Phosphorylase kinase (PhK) is comprised of four different subunits with a stoichiometry of (αβγδ)_4_; α,β, and δ are regulatory, while γ is catalytic. Each of these subunits has isoforms or splice variants differentially expressed in different tissues. Mutations in 4 PhK subunit genes (*PHKA1*, *PHKA2*, *PHKB* and *PHKG2*) have been implicated in low PhK activity in liver and/or muscle [25]–[29]. No variant in the muscle isoform of the PhKγsubunit (*PHKG1*) has been reported for muscle PhK deficiency [17]. To our knowledge, this study is the first one to confirm the association of the *PHKG1* mutation with PhK deficiency, muscle glycogenosis and meat quality traits in pigs.

Pork with high GP (>180* µ*mol/g) often has low ultimate pH and water holding capacity, and is therefore called “acid meat”. The R225Q mutation in *PRKAG3* gene was the first identified causal mutation for acid meat [19]. This gain-of-function mutation causes an increased glucose uptake and glycogen synthesis in skeletal muscle [30], [31]. Its unfavorable allele (225Q) is dominant and specifically present in Hampshire and related breeds [19]. In contrast, the *PHKG1* g.8283C>A mutation that we identified is a loss-of-function mutation causing the defect in glycogen degradation. This variant occurs predominantly in Duroc or Duroc-crossed pigs. The mutant allele *A* appears to be partially recessive, since the average RG value of *AC* heterozygotes approximates to that of *CC* homozygotes rather than *AA* homozygotes (Table S3).

Costa et al. [32] have reported that 9.8% of DLY hybrid pigs free of the *PRKAG3* 225Q allele still show the acid meat phenotype with GP higher than 180* µ*mol/g. In this study, we found that the *PHKG1* QTNs are segregating in DLY hybrid pigs with a frequency of 1.7% (9/540) for the homozygous mutant with GP beyond 180* µ*mol/g. Therefore, we speculate that a substantial proportion of acid meat is likely caused by the *PHKG1* g.8283C>A mutation.

It was observed that the mutation had negative effects on almost all meat quality traits but did not impact any growth traits (e.g. carcass weight; see Table S6) in the F_2_, Sutai and DLY populations. However, Duroc and two Duroc-derived lines carry the mutation at relatively high frequencies (Table 3). The result may suggests that meat quality has not been the primary breeding goals in these pig populations.

In conclusion, we identified a causal mutation in the *PHKG1* gene associated with excess glycogen content in Duroc and its related pigs. The finding highlights the important role of genes encoding PhK subunits in the formation of the GP-related traits. More intriguingly, our finding would be of considerably importance for the pig industry as we can develop a diagnostic DNA test to effectively eliminate the *PHKG1* undesirable allele from nucleus herds and consequently improve meat quality.

## Materials and Methods

### Ethics statement

All procedures involving animals followed the guideline for the care and use of experimental animals established by the Ministry of Agriculture of China. The ethics committee of Jiangxi Agricultural University specifically approved this study.

### Samples and meat quality trait measurement

Three experimental populations were involved in this study: a White Duroc × Erhualian F_2_ intercross, a Chinese Sutai half-sib population and a commercial DLY hybrid population. The F_2_ population was established as described previously [33]. Briefly, two White Duroc boars were mated to 17 Erhualian sows. Nine F_1_ boars and 59 F_1_ sows were then intercrossed to produce a total of 1912 F_2_ animals in 6 batches. Sutai is a Chinese synthetic line that is derived from Chinese Taihu (50%) and Western Duroc (50%) after over 18 generations of artificial selection. The Sutai population comprised offspring of 4 sires and 55 dams. A total of 930 F_2_ individuals, 434 Sutai pigs and 540 DLY pigs were used in this study.

The F_2_ and Sutai pigs were weaned at day 46 and 28 respectively, and males were castrated at day 90 and 18 respectively. The two populations were raised at an experimental farm of Jiangxi Agricultural University in Nanchang city during their fattening period and were slaughtered for phenotype recording at the age of 240±3 days. The DLY pigs grew up at a farm of Xiushui city (about 110 miles away from Nanchang) until the slaughter weight of 90–100kg. All pigs were transported and slaughtered at the same commercial abattoir in Nanchang where the pigs were fasted for 15–20 hours with water available ad libitum. After slaughter, muscle samples were removed within 30 minutes postmortem from the longissimus (LM) and semimembranosus (SM) muscles for RNA isolation and the measurement of meat quality traits. The meat characteristics including the ultimate pH (or pH 24h, measured at 24h postmortem), drip loss, Minolta color parameters (L*, a*, b*), subjective scores of color and marbling, intramuscular fat content (IMF), glycolytic potential (GP), residual glycogen (RG), glucose-6-phosphate (G-6-P) and lactate were measured as described previously [20], [34].

### Array-based SNP genotyping and GWAS mapping

Genomic DNA was isolated from ear, blood or spleen tissues with a standard phenol/chloroform method. A total of 1020 animals from the F_2_ population were genotyped for 62163 SNPs on the Illumina PorcineSNP60 BeadChip [21] according to the standard manufacture's protocol. The 60K Beadchip has 62,163 SNP, of which 54,920 can be mapped to the current pig genome assembly (*Sus Scrofa* Build 10.2) [35]. The quality control (QC) procedures were carried out by Plink v 1.07 [36]. Briefly, animals with call rate>0.9 and Mendelian error rate <0.05, and SNP with call rate>0.9, minor allele frequency>0.05, *P* value>10^−5^ for the Hardy-Weinberg equilibrium test were included. A final set of 39414 informative SNPs on 930 F_2_ pigs were used for subsequent analyses. A total of 434 Sutai pigs were also genotyped using the Illumina PorcineSNP60 BeadChip. To fine map the SSC3 QTL, we increased the marker density in this QTL region with additional 53 SNPs (Table S7), which were identified through comparison of our own whole-genome sequence data from 4 F_0_ Erhualian sows and the Duroc reference genome sequence (*Sus scrofa* Build 10.2). All Sutai pigs were further genotyped for the 53 SNPs by using the *Taq*Man OpenArray Genotyping System (Life Technologies). After QC filtering as described above, 44560 SNPs were included for further analysis. We also genotyped 140 DLY pigs using our developed OpenArray 53-SNP panel. Out of the 53 SNPs, 28 passing QC were included in association analysis. These genotype data are deposited in the Dryad repository (http://dx.doi.org/10.5061/dryad.7kn7r).

The allelic effect of each SNP on phenotypic traits was tested using a general linear mixed model [37]. The model included a random polygenic effect, and the variance-covariance matrix was proportionate to genome-wide identity-by-state [38]. The formula of the model is given in a mathematic expression: 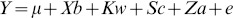, where *Y* is the vector of phenotypes; *µ* is the overall mean; *b* is the vector of fixed effects including sex and batch effects; *w* is the vector of slaughter weight of individuals considered as covariate; *c* is the vector of SNP effects with Erhualian allele substitute to White Duroc allele; *a* is the vector of random additive genetic effects with *α*∼N(0, Gσ_α_^2^), where G is the genomic relationship matrix calculated from the corrected pedigree and σ_α_^2^ is the polygenetic additive variance; *k* is the regression coefficient of slaughter weight and *e* is the vector of residual errors with *e*∼N(0, Iσ_e_^2^), where *I* is the identity matrix and σ_e_^2^ is the residual variance. X, S and Z are incidence matrices for *b*, *w* and *c* respectively. All single-marker GWAS were conducted by GenABEL packages [39].

The genome-wide significance threshold was determined by the Bonferroni method, in which the conventional *P*-value was divided by the number of tests performed [40]. A SNP was considered to have genome-wide significance at *P*<0.05/N and chromosome-wide significance at *P*<1/N, where N is the number of SNPs tested in the analyses. The genome-wide and chromosome-wide significant thresholds were respectively 1.27e-6 (0.05/39414) and 2.54e-5 (1/39414) for the F_2_ population, and 1.12e-6 (0.05/44560) and 2.24e-5 (1/44560) for the Sutai population. The phenotypic variance explained by the top SNPs was estimated by (V_reduce_– V_full_)/V_reduce_, where V_full_ and V_reduce_ are residual variances of models for association analysis with and without SNP term, respectively.

The influence of population stratification was assessed by examining the distribution of test statistics generated from thousands of association tests and assessing their deviation from the null distribution (that expected under the null hypothesis of no SNP associated with the trait) in a quantile-quantile (Q-Q) plot [41]. The Q-Q plot was constructed using R software. Linkage disequilibrium (r^2^) was estimated for SNPs around the QTL region in the two populations by using PLINK v1.07 [36].

### Muscle transcriptome data and eQTL mapping

Total RNA was extracted from longissimus dorsi muscle samples of 497 F_2_ animals using Trizol (Invitrogen). RNA quantity and integrity were assessed using a NanoDrop ND-1000 Spectrophotometer (Thermo Fisher Scientific) and a 2100 Bioanalyser (Agilent). Genome-wide transcripts were assayed by digital gene expression (DGE) system and data processing was conducted as described previously [11], [42]. In brief, the raw tags were first filtered to produce the clean tag data. For mapping clean tags to reference transcript sets or to the pig reference genome (*Sus Scrofa* Build 10.2) [35], we created virtual libraries containing all the possible 17-base length sequences of these resources located next to an *Nla*III restriction site. The reference transcript sets were downloaded from the database of PEDE (Pig Expression Data Explorer; http://pede.dna.affrc.go.jp/) and pig unigene in NCBI (ftp://ftp.ncbi.nih.gov/repository/UniGene/Sus_scrofa/). The redundant transcripts overlapped between the two databases were removed from the reference transcript set. For monitoring the mapping events on both strands, virtual sense and antisense tag sequence databases were generated for both full gene and cDNA sequences using in-house Perl scripts. The clean tag sequences were then mapped using SOAP2 [43] allowing up to one mismatches in 21-bp tag sequences. Sense and antisense tag sequences that unsuccessfully mapped to reference transcripts or mapped to multiple genes were filtered. The number of clean tags that uniquely mapped to the reference transcript sequence of each gene was calculated and then normalized to TPM (number of tags mapped to each gene per million clean tags) as expression level of transcript.

The DGE data of 497 F_2_ animals were adjusted for gender, batch and kinship using a robust linear regression model [44]. Associations between gene expression levels and the RG phenotype were evaluated with Pearson correlation coefficient by R software. eQTL mapping was performed for the DGE profiles in 497 F_2_ animals using mixed linear model implemented by *mmscore* function of GenABEL in R package. Sex and batch were considered as fixed effects, and the genetic co-variances among samples were also taken into account by fitting kinship matrix derived from whole genome SNP genotypes. Bonferroni correction was applied to adjust the multiple tests. All the above mentioned analyses were carried out with R software.

### Resequencing of the *PHKG1* gene

The entire cDNA sequence of *PHKG1* were determined by RT-PCR (reverse transcriptase-polymerase chain reaction) and RACE (rapid amplication of cDNA ends) analysis. First, RNA was extracted from skeletal muscle of 6 F_2_ pigs (2 of each QTL genotype) using Trizol (Invitrogen), then cDNA was synthesized using the PrimerScript RT reagent Kit With gDNA Eraser (Takara) for RT-PCR or the 5′- & 3′- Full Race Kits (Takara) for RACE. The corresponding primer pairs are listed in Table S8. To get full-length gene sequence, we amplified 14 overlapping segments from genomic DNA, using standard PCR condition and primers reported in Table S9. All PCR products were purified using the QIAquick PCR Purification Kit (Qiagen) and sequenced on both strands using the same primers and BigDye Terminator v3.1 Cycle Sequencing kits (Applied Biosystems) and a 3130 DNA Analyzer (Applied Biosystems). The sequence traces were assembled and analyzed for polymorphisms using the SeqMan program (DNASTAR).

### Genotyping of the *PHKG1* g.8283C>A mutation and association analysis

A 140-bp fragment (Figure 3C) encompassing the g.8283C>A mutation was amplified by PCR from genomic DNA using primers (PHKG1-F1: 5′-ATC CCT GTG CTT GCT GGT G-3′; PHKG1-R1: 5′-CCC GGC GGT ACT GGT AAT-3′), digested using enzyme TaqαI and size fractionated by 2% agarose gel electrophoresis. The *A* allele was represented by two fragments of 78 and 62 bp, and the C allele by uncut amplicons.

Association between the *PHKG1* g. 8283C>A polymorphism and meat quality traits was calculated using the least square means method of GLM (General Linear Model) procedure in R software. For the F_2_ and Sutai populations (n = 930 and 434 respectively), sex and batch was included in the model as fix effects, carcass weight as a covariate. A total of 540 DLY pigs were genotyped for this mutation. The residual glycogen content and other meat quality traits were determined for muscle samples from 140 DLY pigs. The model for DLY included sex and harvest batch as fix effects, and carcass weight as a covariate.

The associations between SNP genotypes (i.e. ss131031160 SNP and *PHKG1* g. 8283C>A) and expression levels of three genes (*PHKG1*, *GUSB* and *SUMF2*) were established by One-Way ANOVA analysis in R software. The relationship between the transcript level of *PHKG1* and the RG content was calculated using Pearson correlation in R software.

### Marker-assisted segregation analysis

QTL genotypes of 6 F_1_ boars in the F_2_ population and 3 Sutai F_0_ boars were determined by marker-assisted segregation analysis as described previously [45]. Briefly, a Z-score was calculated for each sire; the score is the log10 of the H_1_/H_0_ likelihood ratio where H_1_ assumes that the boar is heterozygous at the QTL (*Qq*), while H_0_ postulates that the boar is homozygous *QQ* or *qq*. Boars were considered to be *Qq* when Z>2, *QQ* or *qq* when Z <−2, and of undetermined genotype if −2<Z<2.

### Quantitative RT-PCR and western blot analysis

Quantitative RT-PCR (qRT-PCR) reactions were performed in a final volume of 10 µl containing 1 µl of 2.5-fold diluted cDNA (corresponding to 20 ng of starting total RNA), 5 µl Power SYBR Green PCR Master Mix (Applied Biosystems), forward and reverse primers (2 pM each) and 3.6 µl free water. PCRs were conducted on an ABI7900HT instrument (Applied Biosystems) under the following cycling conditions: 10 min at 95°C followed by 40 cycles at 95°C for 15 sec and 60°C for 50 sec. Two primer sets Common-5'-FP/RP and Wt-3'-FP/RP (Figure 4A; Table S10) were used to respectively test total and wild-type transcript (Wt) levels of *PHKG1*. Beta Actin (*ACTB*) was included as endogenous controls. Expression of all assays was measured in triplicates and average values of the triplicates were used for the analysis. The quantification of transcripts was performed by the comparative C_t_(2^-ΔΔCt^) method.

The presence of *PHKG1* Wt transcript in animals with three QTL genotypes was assayed by RT-PCR using the primers 5′-CAC CCC AAC ATC ATA CAG CT-3′ and 5′-ACA GAA GCC AGC ACC GTC-3′ (RT-3'-RP). PCR products of 698-bp were electrophoresed on 1% agarose gel and visualized by UV illumination (Figure 4C).

Total protein from pig muscle tissue was extracted using a Protein Extraction Kit (Applygen). Protein samples separated on SDS-PAGE were transferred onto polyvinylidene difluoride membranes and incubated with rabbit anti-PHKG1 (Proteintech) and rabbit anti-mouse β-Actin (as loading control; Beijing Zhong Shan-Golden Bridge Biological Technology) antibodies. Anti-mouse or anti-rabbit secondary antibodies conjugated with horseradish peroxidase were used and visualized using chemiluminescent substrate (Tiangen Biotech).

### Glycogen phosphorylase kinase (Phk) activity assay

Phk activity of 18 F_2_ animals were determined on their frozen muscle samples using Phk Colorimetric Assay kit (Genmed Scientifics) according to the manufacturer's instructions.

### Splicing minigene reporter assay

#### Generation of minigene constructs

The splicing minigene assay was conducted as previously described [46]. Genomic DNA was isolated from an ear sample of an F_2_ pig with *AA* genotype at the *PHKG1* g.8283C>A mutation site. The *PHKG1* genomic DNA region of 926 bp from partial exon 8 to partial exon 10 was amplified using forward primer (5′-GAC AAG CTT GGA GCA CAG GGG TCA TCA TG-3′) and reverse primer (5′-AGT GGA TCC ACC CTC GCT CCG TTT CTG TG-3′), carrying 5′ tails with *HindIII* and *BamHI* restriction sites (underlined), respectively. After digestion with *HindIII* and *BamHI* enzymes, the PCR products were inserted into the pcDNA3.0 vectors (Invitrogen) with the cytomegalovirus promoter (PCMV). Substitution of adenine (A) to cytosine (C) at the *PHKG1* g.8283C>A site was achieved by site-directed mutagenesis of the mutant expression vector using a QuickChang Site Directed Mutagenesis Kit (Stratagene) with a forward primer (5′-GTT CTC AGG CCC GGA CTC GCG CAG GTG ATC G-3′) and a reverse primer (5′-CAG AGC GAT CAC CTG CGC GAG TCC GGG-3′).

#### Transfection and RT-PCR analysis

The wild-type and mutant minigene constructs were transiently transfected into HeLa cells and 293T cells using Lipofectamine 2000 Transfection Reagent (invitrogen) for 4 h in Opti-MEM (Gibco) medium, according to manufacturer's instructions. Cells were then collected 36 h post-transfection. Total RNA was extracted using TRIzol reagent (Invitrogen). RT-PCR was performed using the PrimerScript RT reagent Kit With gDNA Eraser (Takara), and the forward primer F (5′-GGA AGT GCG TCA CTT CAG C-3′) and the reverse primer R (5′- CGG CGG TAC TGG TAA TAG A-3′). RT-PCR products were separated by electrophoresis on 4% agarose gels containing ethidium bromide and visualized by exposure to ultraviolet light. Each DNA band was gel-extracted using the TIANgel Midi Purification Kit (TIANGEN) and sequenced using the Big Dye Terminator cycle sequencing kit (Applied Biosystems) on a 313XL capillary sequencer (Applied Biosystems). The expression levels of the *PHKG1* minigenes and β-Actin were measured by quantitative real-time RT-PCR on an 7900HT thermal cycler (Applied Biosystems) as described above. The data were analyzed using the comparative C_t_(2^-ΔΔCt^) method.

## Supporting Information

Figure S1 Quantile-quantile plot of SNPs after quality control in genome-wide association analyses for meat quality traits. LM, longissimus muscle; SM, semimembranosus muscle; GP, glycogen potential; pH 24, pH measured at 24h postmortem; 24h-drip loss, drip loss of meat after hanging in an EZ-container (KABE Labortechnik) for 24h; ST, Sutai pigs.
(TIF)

Figure S2 Manhattan plots of genome-wide association analyses for pH 24h and drip loss of semimembranosus muscle from the Sutai population. SM, semimembranosus muscle; ST, Sutai pigs.
(TIF)

Figure S3 The g.8283C>A mutation induces aberrant splicing at the 5′ portion of exon 10 of the *PHKG1* gene. (A) Schematic representation of the *PHKG1* minigenes used in the functional splicing assay, with reference to Gaildrat *et al*. (2010) [46]. (B) RT-PCR analysis of the *PHKG1* spliced transcripts on a 4% agarose gel. RT-PCR products were amplified from total RNA extracted from the HeLa and 293T cells transfected with the wild-type and mutant (g.8283C>A) *PHKG1* minigene constructs. The sizes of the RT-PCR products (78 bp and 62 bp) corresponding to the two mutant transcripts (Mt1 and Mt2) were smaller than that of the RT-PCR product (94 bp) corresponding to the wild-type transcript (Wt). (C) Sequence analysis of the *PHKG1* Wt. Intact segment of exon 10 was observed. (D–E) Sequence analyses of the *PHKG1* Mt1 and Mt2. Two aberrant splicings of the first 16 and 32 nucleotides at 5′ end of exon 10 were observed.
(TIF)

Figure S4 UPGMA tree of 10 kb of the porcine *PHKG1* gene based on 18 sequences classified as representing *q* and *Q* alleles. The tree was constructed using MEGA version 6.06 software developed by Tamura *et al.* (2013) [47] and insertions/deletions were excluded. Bootstrap values (after 1,000 replicates) are reported on the nodes. UPGMA, Unweighted Pair Group Method with Arithmetic Mean.
(TIF)

Figure S5 Haplotype analysis of 18 pig chromosomes with deduced QTL status. These haplotypes consist of 142 polymorphisms (Table S4) in a 10-kb genomic region containing *PHKG1*. The alleles at the polymorphic sites on a *Q* chromosome from the Sutai boar 5675 are indicated as blue blocks, different alleles on other chromosomes as yellow blocks, unclear alleles as grey blocks. The long blue segment correspond to the haplotype shared by all *Q* chromosomes. The QTN is indicated with an asterisk and its allele blocks are highlighted in red on *Q* chromosomes. The chromosomes were originated from Sutai (ST), White Duroc (WD) or Erhualian (Er).
(TIF)

Figure S6 Evaluation of the effects of other SNP on residual glycogen and *PHKG1* gene expression in the Sutai population. (A) When fitting the *PHKG1* g.8283C>A mutation as a cofactor in the model, no other SNP on SSC3 remained significant. (B) No eQTL effect was observed within each group of *PHKG1* genotype *AA*, *AC* and *CC*.
(TIF)

Figure S7 The quantification of mRNA levels from wide-type and mutant *PHKG1* minigenes using real-time RT-PCR. Each minigene was transfected into 293T cells. Total RNA extracted from each cell group was assayed six times. All data were normalized to β-actin mRNA levels, and then expressed as percentage of the wild-type counterpart. The average and standard deviations of expression values obtained from two cell groups with the same minigene are shown.
(TIF)

Figure S8 The strength of associations between residual glycogen (RG) content and the mRNA levels of *GUSB* (r = 0.05, *P* = 0.280; left panel) and *SUMF2* (r = −0.076, *P* = 0.106; right panel) from the DGE data of 412 F_2_ animals.
(TIF)

Figure S9 Regional association plot for residual glycogen content, 24-h drip loss and pH 36h in longissimus muscle from 140 Duroc × (Landrace × Yorkshire) pigs.
(TIF)

Table S1 *PHKG1* gene mutations uncovered by cDNA sequencing.
(DOCX)

Table S2 Consistency between QTL genotypes and SNP genotypes at three sites including the SNP g.8283C>A in *PHKG1* and two GWAS SNPs ss131031160 and ss1315665361 in parental sires from two populations.
(DOCX)

Table S3 Associations of the two GWAS top SNPs (ss131031160 and ss131565361) and the putative QTN g.8283 C>A with residual glycogen in three pig populations.
(DOCX)

Table S4 Identification of *PHKG1* g.8283C>A as a putative QTN through genotype cosegregation analysis in 9 parental boars.
(DOCX)

Table S5 Effect of *PHKG1* g.8283C>A on meat quality traits of longissimus muscle and semimembranosus muscle in Chinese synthetic Sutai pigs.
(DOCX)

Table S6 Effect of *PHKG1* g.8283C>A on carcass weight in White Duroc × Erhualian F_2_ intercross, Sutai pigs and Duroc × (Landrace × Yorkshire) hybrid pigs.
(DOCX)

Table S7 The 53 SNPs detected by OpenArray genotyping platform.
(DOCX)

Table S8 Primers used for cDNA sequencing of the *PHKG1* gene.
(DOCX)

Table S9 Primers used for DNA sequencing of the *PHKG1* gene.
(DOCX)

Table S10 Primers used for quantitative RT-PCR.
(DOCX)
