# Supplementary figures and images for: A Splice Mutation in the PHKG1 Gene Causes High Glycogen Content and Low Meat Quality in Pig Skeletal Muscle
Source: PLoS Genet. 2014 Oct 23;10(10):e1004710. doi: 10.1371/journal.pgen.1004710 (PMC4207639; doi:10.1371/journal.pgen.1004710)

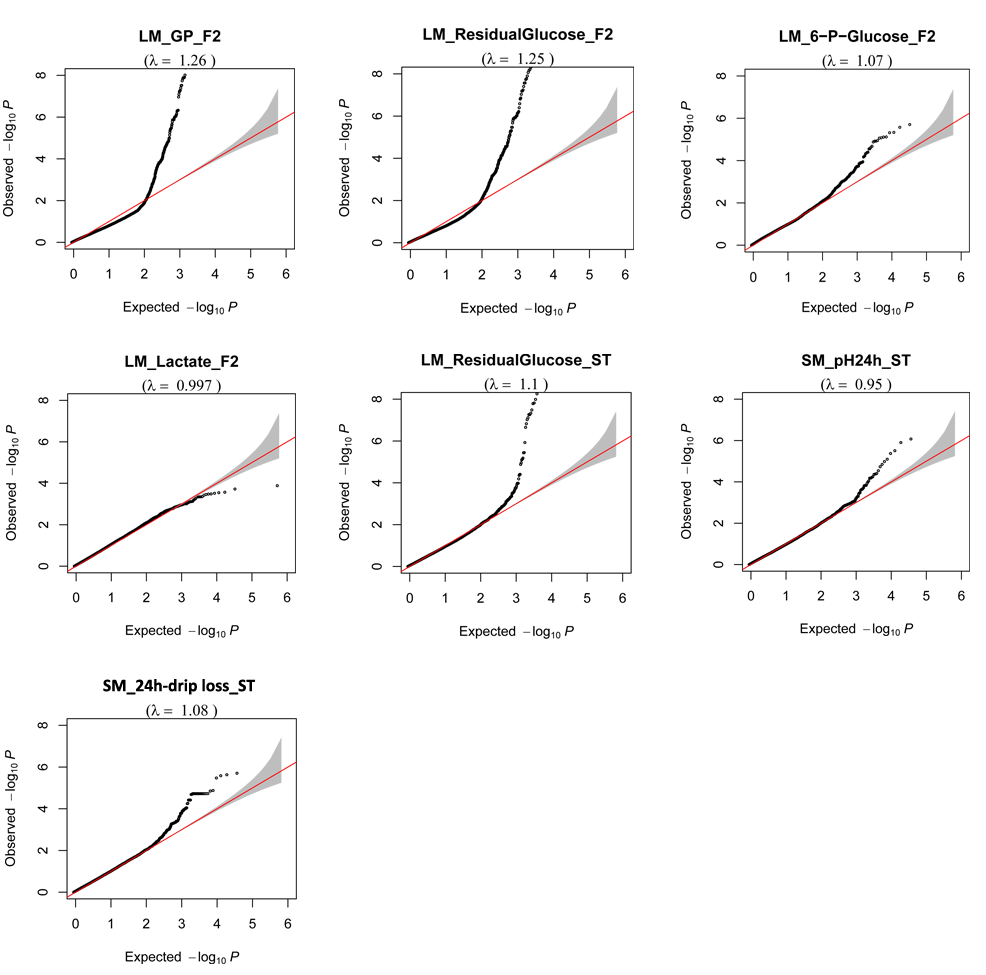

Supplement: Figure S1 — Quantile-quantile plot of SNPs after quality control in genome-wide association analyses for meat quality traits. LM, longissimus muscle; SM, semimembranosus muscle; GP, glycogen potential; pH 24, pH measured at 24h postmortem; 24h-drip loss, drip loss of meat after hanging in an EZ-container (KABE Labortechnik) for 24h; ST, Sutai pigs. (TIF) [file pgen.1004710.s001.tif]

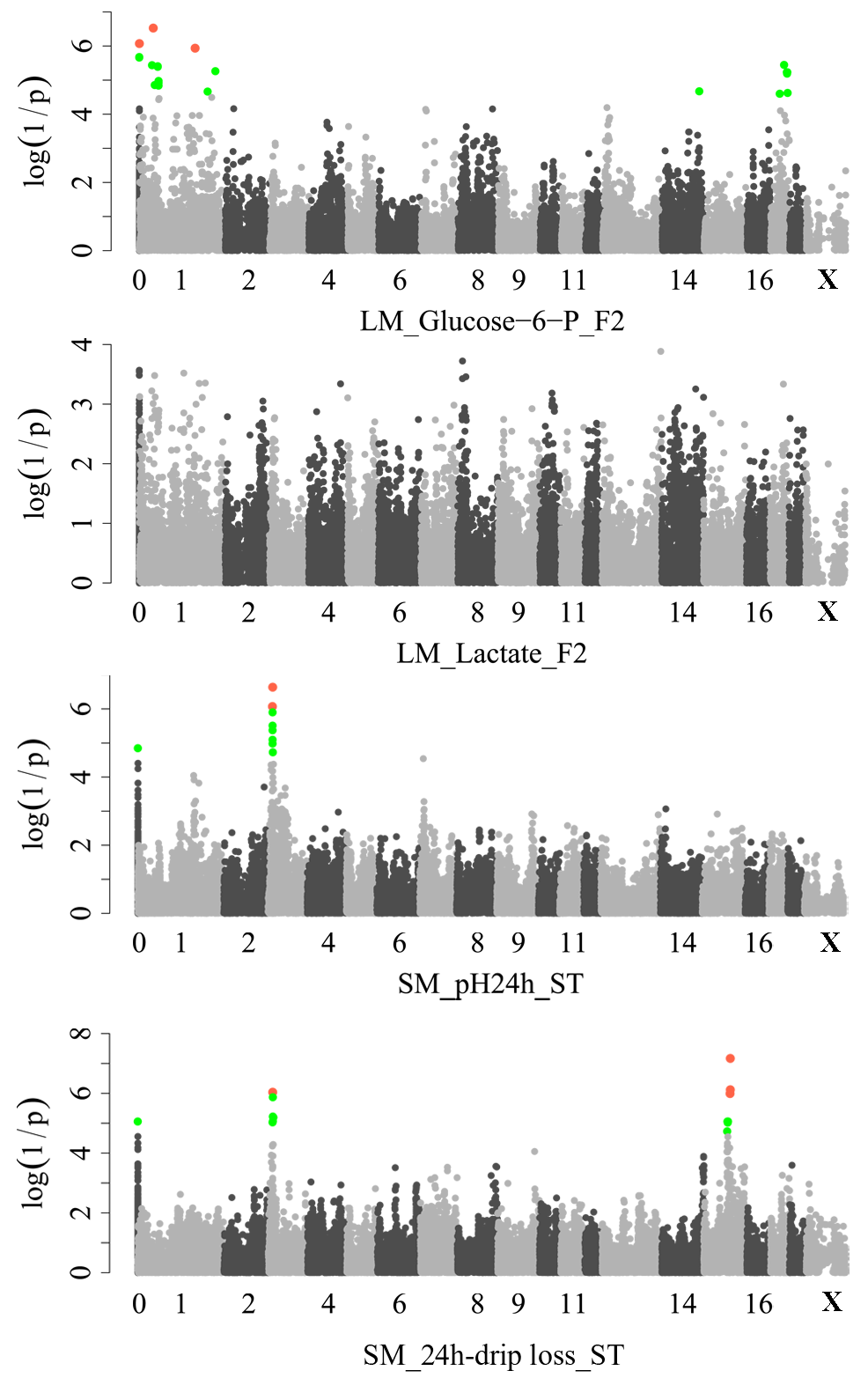

Supplement: Figure S2 — Manhattan plots of genome-wide association analyses for pH 24h and drip loss of semimembranosus muscle from the Sutai population. SM, semimembranosus muscle; ST, Sutai pigs. (TIF) [file pgen.1004710.s002.tif]

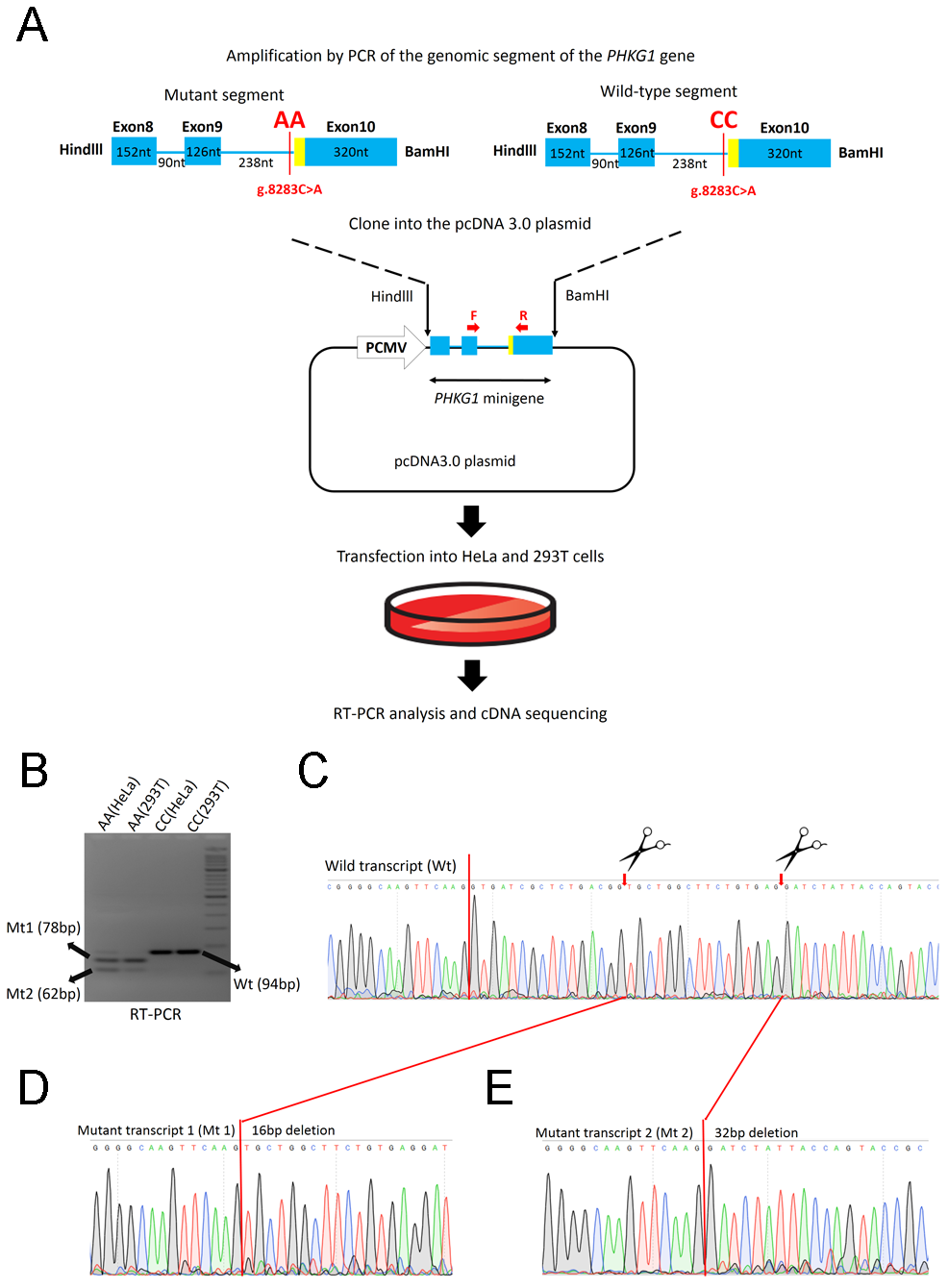

Supplement: Figure S3 — The g.8283C>A mutation induces aberrant splicing at the 5′ portion of exon 10 of the PHKG1 gene. (A) Schematic representation of the PHKG1 minigenes used in the functional splicing assay, with reference to Gaildrat et al. (2010) [46]. (B) RT-PCR analysis of the PHKG1 spliced transcripts on a 4% agarose gel. RT-PCR products were amplified from total RNA extracted from the HeLa and 293T cells transfected with the wild-type and mutant (g.8283C>A) PHKG1 minigene constructs. The sizes of the RT-PCR products (78 bp and 62 bp) corresponding to the two mutant transcripts (Mt1 and Mt2) were smaller than that of the RT-PCR product (94 bp) corresponding to the wild-type transcript (Wt). (C) Sequence analysis of the PHKG1 Wt. Intact segment of exon 10 was observed. (D–E) Sequence analyses of the PHKG1 Mt1 and Mt2. Two aberrant splicings of the first 16 and 32 nucleotides at 5′ end of exon 10 were observed. (TIF) [file pgen.1004710.s003.tif]

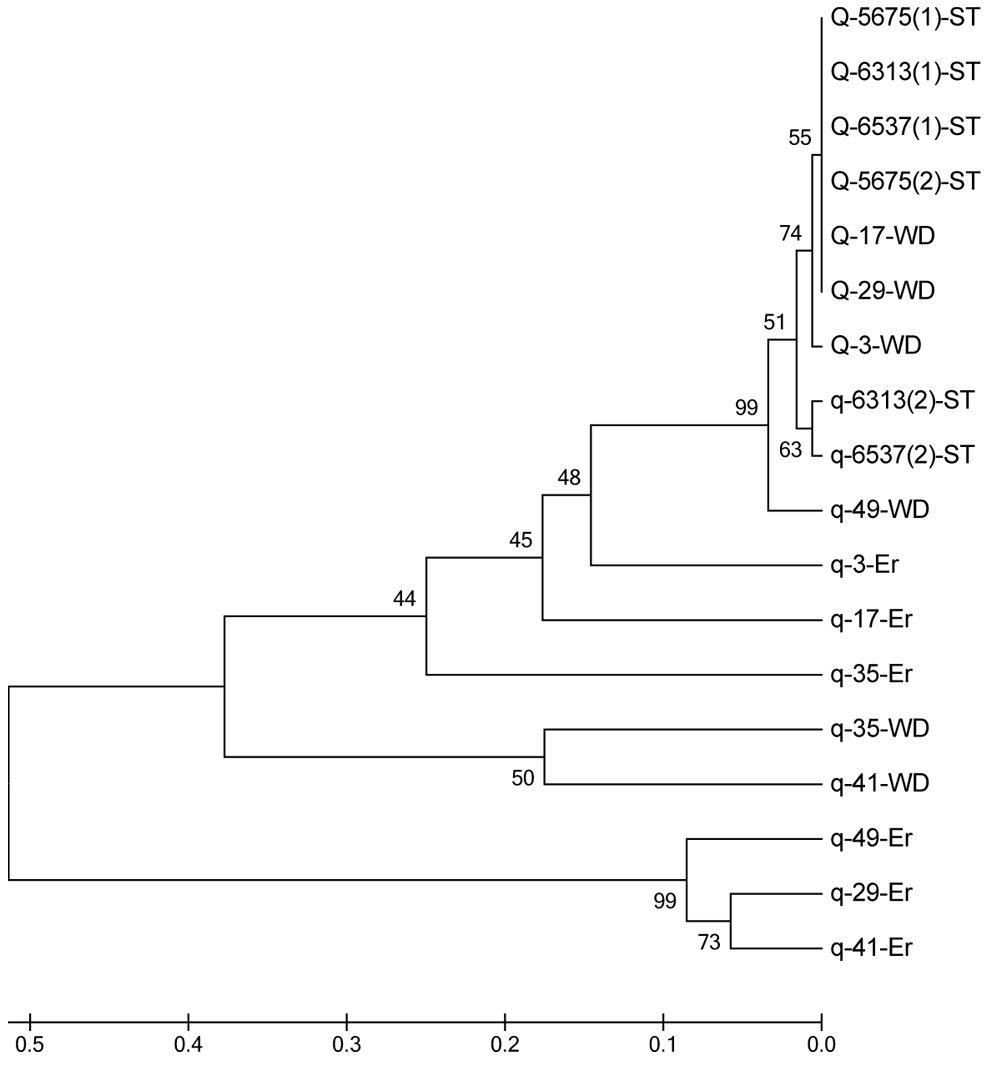

Supplement: Figure S4 — UPGMA tree of 10 kb of the porcine PHKG1 gene based on 18 sequences classified as representing q and Q alleles. The tree was constructed using MEGA version 6.06 software developed by Tamura et al. (2013) [47] and insertions/deletions were excluded. Bootstrap values (after 1,000 replicates) are reported on the nodes. UPGMA, Unweighted Pair Group Method with Arithmetic Mean. (TIF) [file pgen.1004710.s004.tif]

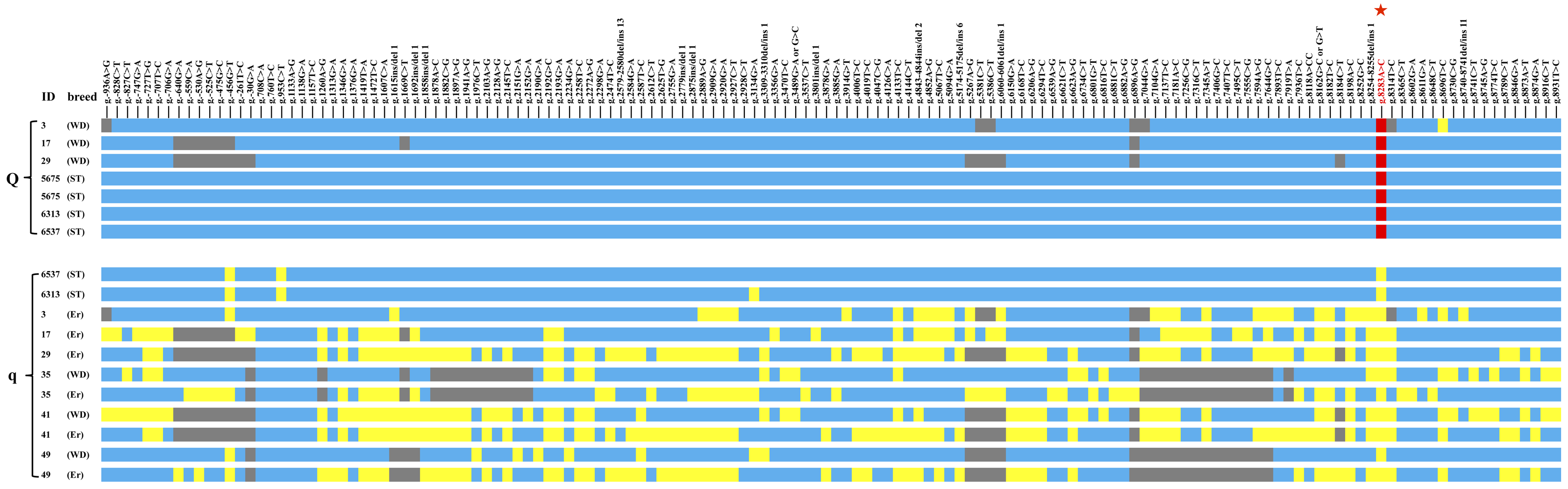

Supplement: Figure S5 — Haplotype analysis of 18 pig chromosomes with deduced QTL status. These haplotypes consist of 142 polymorphisms (Table S4) in a 10-kb genomic region containing PHKG1. The alleles at the polymorphic sites on a Q chromosome from the Sutai boar 5675 are indicated as blue blocks, different alleles on other chromosomes as yellow blocks, unclear alleles as grey blocks. The long blue segment correspond to the haplotype shared by all Q chromosomes. The QTN is indicated with an asterisk and its allele blocks are highlighted in red on Q chromosomes. The chromosomes were originated from Sutai (ST), White Duroc (WD) or Erhualian (Er). (TIF) [file pgen.1004710.s005.tif]

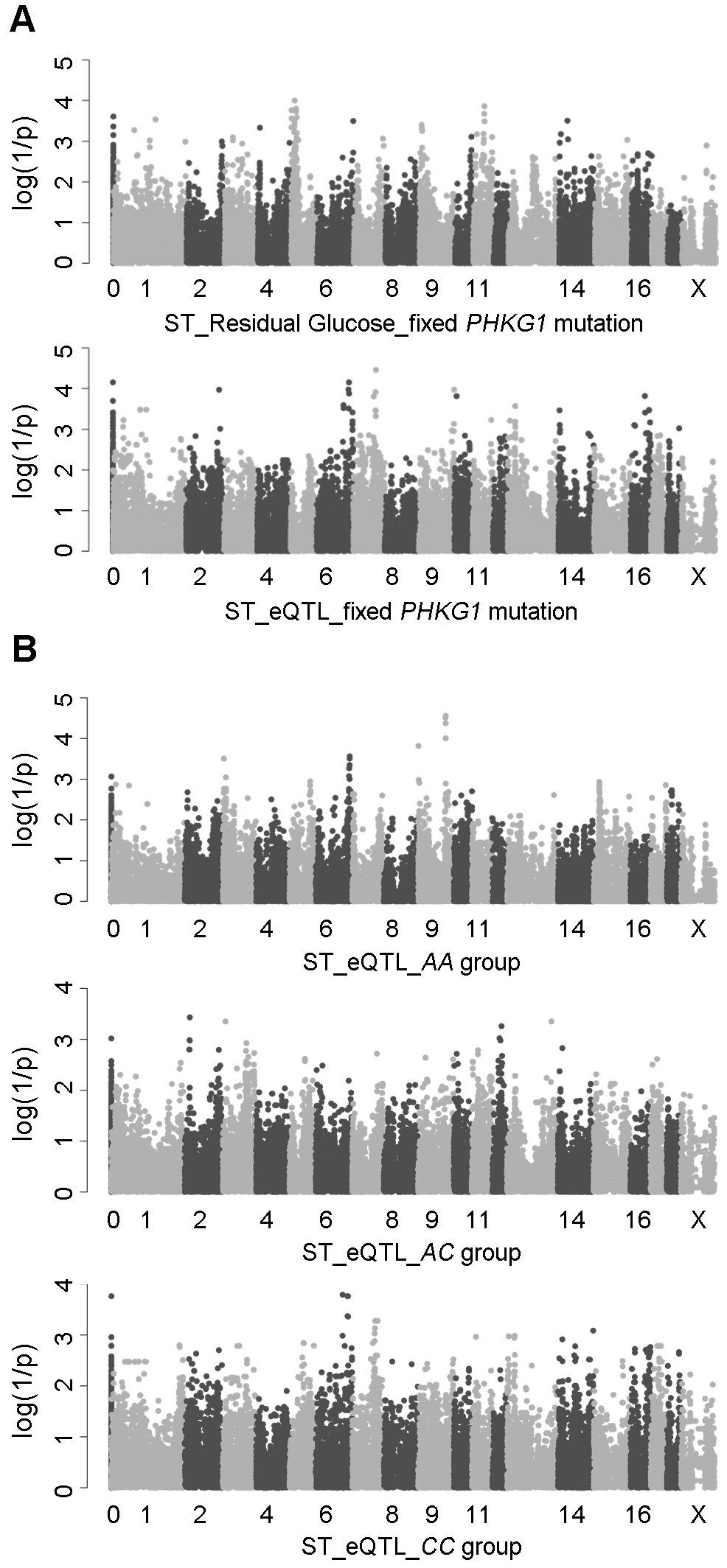

Supplement: Figure S6 — Evaluation of the effects of other SNP on residual glycogen and PHKG1 gene expression in the Sutai population. (A) When fitting the PHKG1 g.8283C>A mutation as a cofactor in the model, no other SNP on SSC3 remained significant. (B) No eQTL effect was observed within each group of PHKG1 genotype AA, AC and CC. (TIF) [file pgen.1004710.s006.tif]

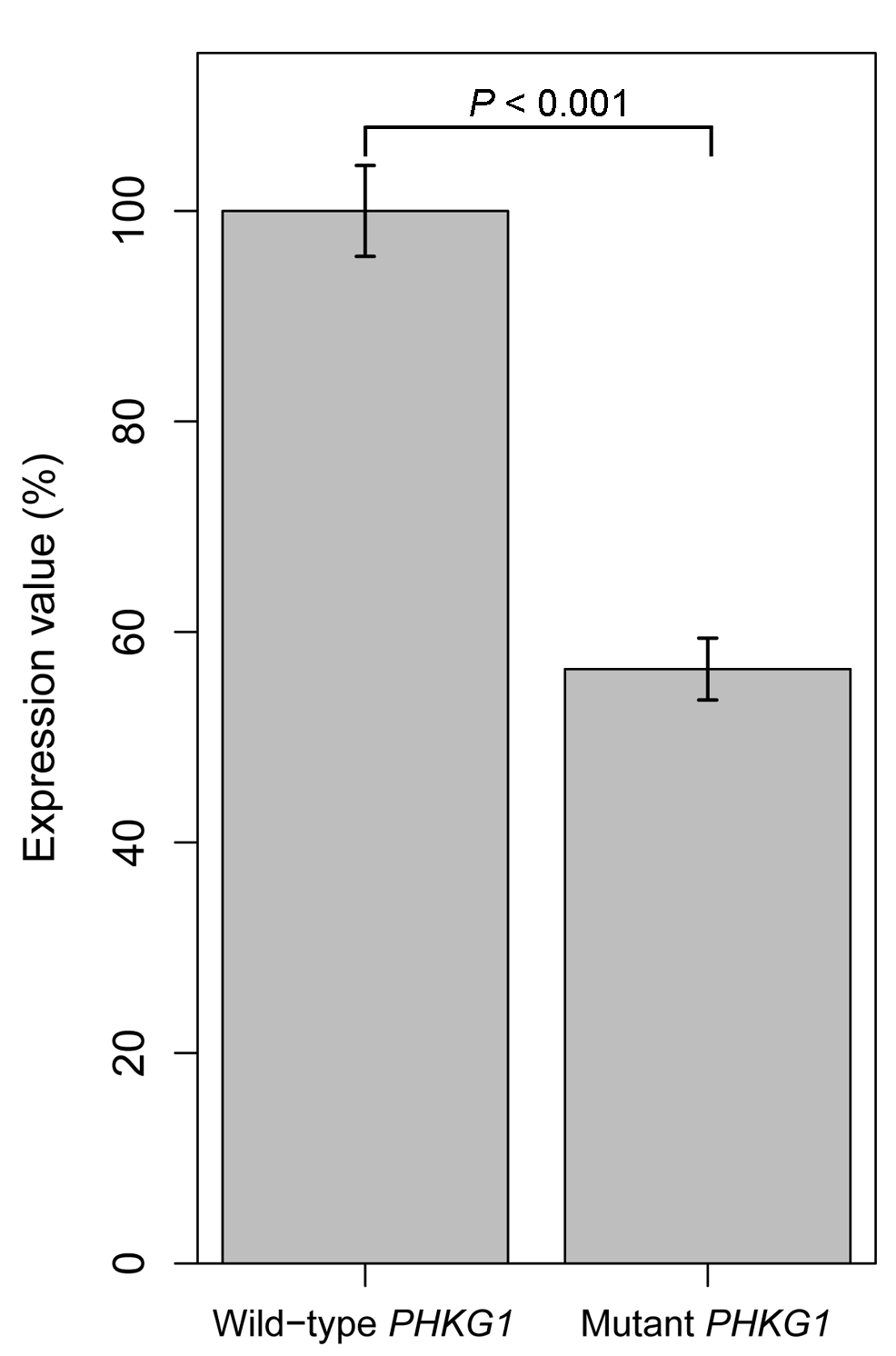

Supplement: Figure S7 — The quantification of mRNA levels from wide-type and mutant PHKG1 minigenes using real-time RT-PCR. Each minigene was transfected into 293T cells. Total RNA extracted from each cell group was assayed six times. All data were normalized to β-actin mRNA levels, and then expressed as percentage of the wild-type counterpart. The average and standard deviations of expression values obtained from two cell groups with the same minigene are shown. (TIF) [file pgen.1004710.s007.tif]

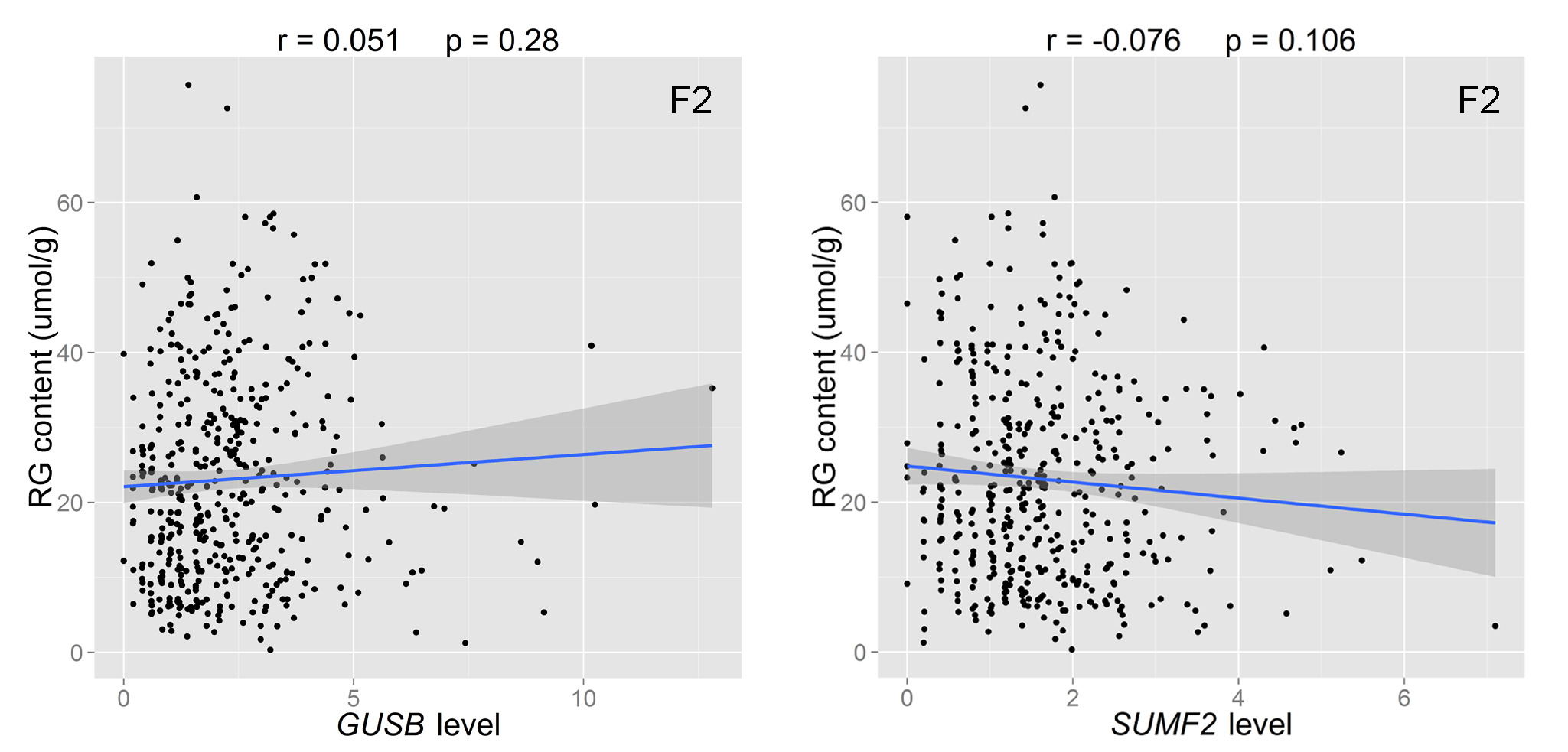

Supplement: Figure S8 — The strength of associations between residual glycogen (RG) content and the mRNA levels of GUSB (r = 0.05, P = 0.280; left panel) and SUMF2 (r = −0.076, P = 0.106; right panel) from the DGE data of 412 F2 animals. (TIF) [file pgen.1004710.s008.tif]

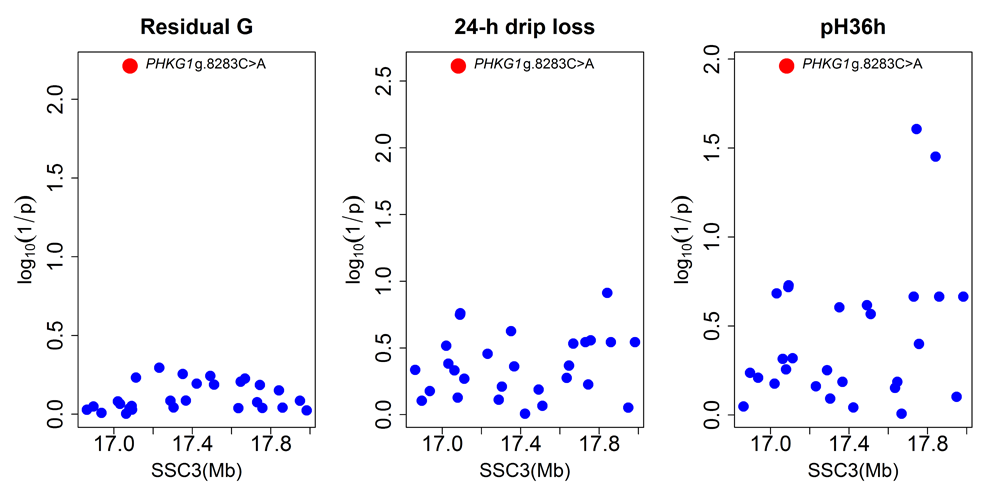

Supplement: Figure S9 — Regional association plot for residual glycogen content, 24-h drip loss and pH 36h in longissimus muscle from 140 Duroc × (Landrace × Yorkshire) pigs. (TIF) [file pgen.1004710.s009.tif]
