# Supplementary material for: A Splice Mutation in the PHKG1 Gene Causes High Glycogen Content and Low Meat Quality in Pig Skeletal Muscle
Source: PLoS Genet. 2014 Oct 23;10(10):e1004710. doi: 10.1371/journal.pgen.1004710 (PMC4207639; doi:10.1371/journal.pgen.1004710)
Supplement: Table S1 — PHKG1 gene mutations uncovered by cDNA sequencing. (DOCX) [file pgen.1004710.s010.docx]

**Table S1.** *PHKG1* mutations uncovered by cDNA sequencing

| No. | Mutation  （chr: position^a^, bp） | Exon | CDS or UTR^b^ | Mutation type |
| --- | --- | --- | --- | --- |
| 1 | c.A>G (3:17077917) | 3 | CDS | synonymous |
| 2 | c.C>T (3:17077929) | 3 | CDS | synonymous |
| 3 | c.A>G (3:17080878) | 6 | CDS | synonymous |
| 4 | c.C>T (3:17082148) | 10 | CDS | synonymous |
| 5 | c.del/ins32 (3:17082122-17082153) | 10 | CDS | deletion/insertion |
| 6 | c.G>A (3:17082444) | 10 | 3’-UTR | — |
| 7 | c.C>T (3:17082530) | 10 | 3’-UTR | — |
| 8 | c.C>G (3:17082564) | 10 | 3’-UTR | — |
| 9 | c.del/ins10 (3:17082564-17082574) | 10 | 3’-UTR | — |
| 10 | c.C>T (3:17082575) | 10 | 3’-UTR | — |
| 11 | c.A>G (3:17082579) | 10 | 3’-UTR | — |
| 12 | c.C>T (3:17082608) | 10 | 3’-UTR | — |
| 13 | c.A>T (3:17082707) | 10 | 3’-UTR | — |
| 14 | c.C>T (3:17082765) | 10 | 3’-UTR | — |

^a^the location of the mutation on Sus scrofa genome assembly 10.2 (Sscrofa10.2)

^b^CDS, coding sequence; UTR, untranslated region.
