# Supplementary material for: A Splice Mutation in the PHKG1 Gene Causes High Glycogen Content and Low Meat Quality in Pig Skeletal Muscle
Source: PLoS Genet. 2014 Oct 23;10(10):e1004710. doi: 10.1371/journal.pgen.1004710 (PMC4207639; doi:10.1371/journal.pgen.1004710)
Supplement: Table S2 — Consistency between QTL genotypes and SNP genotypes at three sites including the SNP g.8283C>A in PHKG1 and two GWAS SNPs ss131031160 and ss1315665361 in parental sires from two populations. (DOCX) [file pgen.1004710.s011.docx]

**Table S2.** Consistency between QTL genotypes and SNP genotypes at three sites including the SNP g.8283C>A in *PHKG1* and two GWAS SNPs ss131031160 and ss1315665361 in parental sires from two populations.

| Populations^a^ | Generation | Animal ID | *Z* score | QTL genotypes^b^ | *PHKG1* g.8283C>A | ss131031160 | ss1315665361 |
| --- | --- | --- | --- | --- | --- | --- | --- |
| WE | F_1_ | 3 | 2.20 | Qq | AC | AG | AG |
|  |  | 17 | 3.01 | Qq | AC | AG | AG |
|  |  | 29 | 2.69 | Qq | AC | AG | AG |
|  |  | 35 | -3.08 | qq/QQ | CC | GG | AG^e^ |
|  |  | 41 | -2.00 | qq/QQ | CC | GG | GG |
|  |  | 49 | -3.26 | qq/QQ | CC | GG | AG^e^ |
| ST | F_0_ | 5675 | *-1.57* | *qq/QQ* | AA | AA | AA |
|  |  | 6313 | *1.71* | *Qq* | AC | AG | AA^e^ |
|  |  | 6537 | 2.93 | Qq | AC | AA^e^ | AG |

^a^ WE, White Duroc × Erhualian F_2_ intercross; ST, Sutai pigs;

^b^ The italic Z scores for two boars 5675 and 6313 are close to -2 or 2, so it is high likely that they were respectively homozygous and heterozygous for the QTL affecting glycolytic potential.

^c,d^ ss131031160 and ss1315665361 were the top SNPs associated with glycolytic potential identified by GWAS in the White Duroc × Erhualian F_2_ intercross and Sutai pigs, respectively.

^e^ SNP genotypes underlined are not consistent with QTL genotypes.
