# Supplementary material for: A Splice Mutation in the PHKG1 Gene Causes High Glycogen Content and Low Meat Quality in Pig Skeletal Muscle
Source: PLoS Genet. 2014 Oct 23;10(10):e1004710. doi: 10.1371/journal.pgen.1004710 (PMC4207639; doi:10.1371/journal.pgen.1004710)
Supplement: Table S3 — Associations of the two GWAS top SNPs (ss131031160 and ss131565361) and the putative QTN g.8283 C>A with residual glycogen in three pig populations. (DOCX) [file pgen.1004710.s012.docx]

**Table S3.** Associations of the two GWAS top SNPs (ss131031160 and ss131565361) and the putative QTN g.8283 C>A with residual glycogen in three pig populations.

|  |  | White Duroc × Erhualian F2 pigs | | |  | Sutai pigs | | |  | Duroc × (Landrace × Yorkshire) pigs | | |
| --- | --- | --- | --- | --- | --- | --- | --- | --- | --- | --- | --- | --- |
| **SNP** | **Genotype** | **N** | **RG (µmol/g)^a^** | ***P* value** |  | **N** | **RG (µmol/g)** | ***P* value** |  | **N** | **RG (µmol/g)** | ***P* value** |
| ss131031160 | AA | 45 | 46.75±11.90 | 2.00E-34 |  | 139 | 36.55±18.57 | 7.32E-32 |  | 47 | 13.21±8.50 | 0.961 |
|  | AG | 344 | 24.95±13.68 |  |  | 158 | 16.17±13.69 |  |  | 61 | 12.68±11.27 |  |
|  | GG | 475 | 20.99±12.25 |  |  | 74 | 12.52±11.18 |  |  | 23 | 12.61±12.83 |  |
| ss131565361 | AA | 144 | 32.00±16.49 | 4.22E-15 |  | 149 | 39.15±22.04 | 6.53E-42 |  | 91 | 13.41±11.08 | 0.419 |
|  | AG | 431 | 23.33±12.91 |  |  | 207 | 16.38±13.05 |  |  | 31 | 12.49±9.79 |  |
|  | GG | 284 | 20.71±12.58 |  |  | 74 | 9.19±7.38 |  |  | 9 | 8.57±7.58 |  |
| g.8283 C>A | AA | 45 | 46.75±11.90 | **2.00E-34** |  | 110 | 44.60±19.35 | **9.03E-54** |  | 9 | 20.84±11.50 | **0.006** |
|  | AC | 344 | 24.95±13.68 |  |  | 204 | 15.91±13.34 |  |  | 46 | 16.54±12.44 |  |
|  | CC | 475 | 20.99±12.25 |  |  | 105 | 11.74±10.39 |  |  | 85 | 11.04±8.36 |  |

^a^Mean±S.D. of residual glycogen (RG) in longissimus muscle.
