# Supplementary material for: A Splice Mutation in the PHKG1 Gene Causes High Glycogen Content and Low Meat Quality in Pig Skeletal Muscle
Source: PLoS Genet. 2014 Oct 23;10(10):e1004710. doi: 10.1371/journal.pgen.1004710 (PMC4207639; doi:10.1371/journal.pgen.1004710)
Supplement: Table S4 — Identification of PHKG1 g.8283C>A as a putative QTN through genotype cosegregation analysis in 9 parental boars. (DOCX) [file pgen.1004710.s013.docx]

**Table S4**. Identification of *PHKG1* g.8283C>A as a putative QTN through genotype cosegregation analysis in 9 parental boars^a^.

|  |  |  |  | White Duroc × Erhualian F_1_ boars | | | | | |  | Sutai F_0_ boars | | |
| --- | --- | --- | --- | --- | --- | --- | --- | --- | --- | --- | --- | --- | --- |
| No. | Location | Mutations | Chr: position, bp | 3  (Qq) | 17  (Qq) | 29  (Qq) | 35  (qq/QQ) | 41  (qq/QQ) | 49  (qq/QQ) |  | 5675  (qq/QQ) | 6313  (Qq) | 6537  (Qq) |
| 1 | Upstream | g.-936A>G | 3:17073848 | — | AG | *AA* | AA | *AG* | AA |  | AA | *AA* | *AA* |
| 2 |  | g.-828C>T | 3:17073956 | *CC* | CT | *CC* | CC | *CT* | CC |  | CC | *CC* | *CC* |
| 3 |  | g.-827C>T | 3:17073957 | *CC* | *CC* | *CC* | *CT* | *CT* | CC |  | CC | *CC* | *CC* |
| 4 |  | g.-747G>A | 3:17074037 | *GG* | GA | *GG* | GG | *GA* | GG |  | GG | *GG* | *GG* |
| 5 |  | g.-727T>G | 3:17074057 | *TT* | TG | TG | *TG* | GG | TT |  | TT | *TT* | *TT* |
| 6 |  | g.-707T>C | 3:17074077 | *TT* | TC | TC | *TC* | CC | TT |  | TT | *TT* | *TT* |
| 7 |  | g.-706G>A | 3:17074078 | *GG* | GA | *GG* | GG | *GA* | GG |  | GG | *GG* | *GG* |
| 8 |  | g.-640G>A | 3:17074144 | *GG* | — | — | GG | — | *GA* |  | GG | *GG* | *GG* |
| 9 |  | g.-559C>A | 3:17074225 | *CC* | — | — | *CA* | — | CC |  | CC | *CC* | *CC* |
| 10 |  | g.-530A>G | 3:17074254 | *AA* | — | — | *AG* | — | *AG* |  | AA | *AA* | *AA* |
| 11 |  | g.-525C>T | 3:17074259 | *CC* | — | — | *CT* | — | CC |  | CC | *CC* | *CC* |
| 12 |  | g.-475G>C | 3:17074309 | *GG* | — | — | *GC* | — | GG |  | GG | *GG* | *GG* |
| 13 |  | g.-456G>T | 3:17074328 | GT | — | — | *GT* | — | TT |  | GG | GT | GT |
| 14 |  | g.-261T>C | 3:17074523 | *TT* | TC | — | TT | — | TT |  | TT | *TT* | *TT* |
| 15 |  | g.-30G>A | 3:17074754 | *GG* | GA | — | — | — | *GA* |  | GG | *GG* | *GG* |
| 16 | Intron1 | g.708C>A | 3:17075491 | *CC* | *CC* | *CC* | CC | AA | *CA* |  | CC | *CC* | *CC* |
| 17 |  | g.760T>C | 3:17075543 | *TT* | *TT* | *TT* | TT | CC | *TC* |  | TT | *TT* | *TT* |
| 18 |  | g.953C>T | 3:17075736 | *CC* | *CC* | *CC* | CC | CC | *CT* |  | CC | CT | CT |
| 19 |  | g.1133A>G | 3:17075916 | *AA* | *AA* | *AA* | AA | GG | *AG* |  | AA | *AA* | *AA* |
| 20 |  | g.1138G>A | 3:17075921 | *GG* | *GG* | *GG* | GG | GG | *GA* |  | GG | *GG* | *GG* |
| 21 |  | g.1157T>C | 3:17075939 | *TT* | *TT* | *TT* | TT | CC | *TC* |  | TT | *TT* | *TT* |
| 22 |  | g.1260A>G | 3:17076043 | *AA* | — | AG | — | GG | — |  | AA | *AA* | *AA* |
| 23 |  | g.1313G>A | 3:17076096 | *GG* | *GG* | *GG* | GG | GG | *GA* |  | GG | *GG* | *GG* |
| 24 |  | g.1346G>A | 3:17076129 | *GG* | GA | GA | *GA* | AA | *GA* |  | GG | *GG* | *GG* |
| 25 |  | g.1376G>A | 3:17076159 | *GG* | *GG* | *GG* | GG | *GA* | GG |  | GG | *GG* | *GG* |
| 26 |  | g.1419T>A | 3:17076202 | *TT* | TA | TA | *TA* | AA | *TA* |  | TT | *TT* | *TT* |
| 27 |  | g.1472T>C | 3:17076255 | *TT* | TC | TC | *TC* | CC | *TC* |  | TT | *TT* | *TT* |
| 28 |  | g.1607C>A | 3:17076390 | *CC* | CA | CA | *CA* | AA | *CA* |  | CC | *CC* | *CC* |
| 29 |  | g.1615ins/del 1 | 3:17076399 | ins/del | ins/del | ins/del | *ins/del* | del/del | — |  | ins/ins | *ins/ins* | *ins/ins* |
| 30 |  | g.1669C>T | 3:17076452 | *CC* | — | CT | — | TT | — |  | CC | *CC* | *CC* |
| 31 |  | g.1692ins/del 1 | 3:17076475 | *ins/ins* | ins/del | ins/del | *ins/del* | del/del | — |  | ins/ins | *ins/ins* | *ins/ins* |
| 32 |  | g.1858ins/del 1 | 3:17076641 | *ins/ins* | *ins/ins* | ins/del | ins/ins | del/del | *ins/del* |  | ins/ins | *ins/ins* | *ins/ins* |
| 33 |  | g.1878A>C | 3:17076661 | *AA* | *AA* | AC | — | CC | *AC* |  | AA | *AA* | *AA* |
| 34 |  | g.1882C>G | 3:17076665 | *CC* | *CC* | CG | — | GG | *CG* |  | CC | *CC* | *CC* |
| 35 |  | g.1897A>G | 3:17076680 | *AA* | *AA* | AG | — | GG | *AG* |  | AA | *AA* | *AA* |
| 36 |  | g.1941A>G | 3:17076724 | *AA* | *AA* | AG | — | GG | *AG* |  | AA | *AA* | *AA* |
| 37 |  | g.1976C>T | 3:17076759 | *CC* | *CC* | *CC* | — | CC | *CT* |  | CC | *CC* | *CC* |
| 38 |  | g.2103A>G | 3:17076886 | *AA* | *AA* | AG | — | GG | *AG* |  | AA | *AA* | *AA* |
| 39 |  | g.2128A>G | 3:17076911 | *AA* | *AA* | *AA* | — | *AG* | AA |  | AA | *AA* | *AA* |
| 40 |  | g.2145T>C | 3:17076928 | *TT* | *TT* | TC | — | CC | TT |  | TT | *TT* | *TT* |
| 41 |  | g.2151G>A | 3:17076934 | *GG* | *GG* | *GG* | — | GG | *GA* |  | GG | *GG* | *GG* |
| 42 |  | g.2152G>A | 3:17076935 | *GG* | *GG* | *GG* | — | *GA* | GG |  | GG | *GG* | *GG* |
| 43 |  | g.2190G>A | 3:17076973 | *GG* | *GG* | *GG* | GG | GG | *GA* |  | GG | *GG* | *GG* |
| 44 |  | g.2192G>C | 3:17076975 | *GG* | GC | GC | *GC* | CC | GG |  | GG | *GG* | *GG* |
| 45 |  | g.2193G>A | 3:17076976 | *GG* | GA | GA | *GA* | AA | GG |  | GG | *GG* | *GG* |
| 46 |  | g.2234G>A | 3:17077017 | *GG* | *GG* | *GG* | GG | GG | *GA* |  | GG | *GG* | *GG* |
| 47 |  | g.2258T>C | 3:17077041 | *TT* | *TT* | TC | *TC* | CC | *TC* |  | TT | *TT* | *TT* |
| 48 |  | g.2272A>G | 3:17077055 | *AA* | *AA* | AG | *AG* | GG | *AG* |  | AA | *AA* | *AA* |
| 49 |  | g.2298G>A | 3:17077081 | *GG* | *GG* | *GG* | *GA* | GG | GG |  | GG | *GG* | *GG* |
| 50 | Exon2 | g.2474T>C | 3:17077257 | *TT* | *TT* | TC | *TC* | *TC* | *TC* |  | TT | *TT* | *TT* |
| 51 | Intron2 | g.2579-2580del/ins 13 | 3:17077362-17077363 | *del/del* | *del/del* | del/ins | del/del | ins/ins | *del/ins* |  | del/del | *del/del* | *del/del* |
| 52 |  | g.2584G>A | 3:17077367 | *GG* | *GG* | GA | GG | *GA* | *GA* |  | GG | *GG* | *GG* |
| 53 |  | g.2587T>C | 3:17077370 | *TT* | *TT* | TC | TT | CC | CC |  | TT | *TT* | *TT* |
| 54 |  | g.2612C>T | 3:17077395 | *CC* | *CC* | *CC* | *CT* | *CT* | CC |  | CC | *CC* | *CC* |
| 55 |  | g.2625T>G | 3:17077408 | *TT* | *TT* | TG | TT | *TG* | *TG* |  | TT | *TT* | *TT* |
| 56 |  | g.2755G>A | 3:17077538 | GA | *GG* | GA | GG | *GA* | *GA* |  | GG | *GG* | *GG* |
| 57 |  | g.2779ins/del 1 | 3:17077562 | ins/del | *ins/ins* | ins/del | ins/ins | *ins/del* | *ins/del* |  | ins/ins | *ins/ins* | *ins/ins* |
| 58 |  | g.2875ins/del 1 | 3:17077658 | ins/del | *ins/ins* | ins/del | *ins/del* | *ins/del* | *ins/del* |  | ins/ins | *ins/ins* | *ins/ins* |
| 59 |  | g.2889A>G | 3:17077672 | AG | *AA* | AG | *AG* | *AG* | *AG* |  | AA | *AA* | *AA* |
| 60 |  | g.2909G>A | 3:17077692 | GA | *GG* | GA | *GA* | *GA* | *GA* |  | GG | *GG* | *GG* |
| 61 |  | g.2920G>A | 3:17077703 | GA | *GG* | GA | *GA* | *GA* | *GA* |  | GG | *GG* | *GG* |
| 62 |  | g.2927C>T | 3:17077710 | CT | *CC* | CT | *CT* | *CT* | *CT* |  | CC | *CC* | *CC* |
| 63 |  | g.2928C>T | 3:17077711 | *CC* | *CC* | *CC* | *CT* | CC | CC |  | CC | *CC* | *CC* |
| 64 | Exon3 | g.3134G>A | 3:17077917 | *GG* | *GG* | *GG* | *GA* | GG | *GA* |  | GG | GA | *GG* |
| 65 | Intron3 | g.3309-3310del/ins 1 | 3:17078093 | *ins/ins* | *ins/ins* | del/ins | *del/ins* | *del/ins* | *del/ins* |  | ins/ins | *ins/ins* | *ins/ins* |
| 66 |  | g.3356G>A | 3:17078139 | *GG* | GA | *GG* | GG | GG | GG |  | GG | *GG* | *GG* |
| 67 |  | g.3470T>C | 3:17078253 | *TT* | *TT* | *TT* | *TC* | *TC* | TT |  | TT | *TT* | *TT* |
| 68 |  | g.3489G>A or G>C | 3:17078272 | *GG* | *GG* | *GG* | *GA* | *GC* | GG |  | GG | *GG* | *GG* |
| 69 |  | g.3537C>T | 3:17078320 | *CC* | *CC* | *CC* | *CT* | CC | CC |  | CC | *CC* | *CC* |
| 70 | Intron4 | g.3801ins/del 1 | 3:17078584 | *ins/ins* | ins/del | *ins/ins* | ins/ins | ins/ins | ins/ins |  | ins/ins | *ins/ins* | *ins/ins* |
| 71 |  | g.3878G>A | 3:17078661 | *GG* | *GG* | GA | GG | *GA* | *GA* |  | GG | *GG* | *GG* |
| 72 |  | g.3885G>A | 3:17078668 | *GG* | *GG* | *GG* | *GA* | GG | GG |  | GG | *GG* | *GG* |
| 73 |  | g.3914G>T | 3:17078697 | GT | *GG* | *GG* | GG | GG | GG |  | GG | *GG* | *GG* |
| 74 |  | g.4006T>C | 3:17078789 | *TT* | *TT* | TC | TT | *TC* | *TC* |  | TT | *TT* | *TT* |
| 75 |  | g.4019T>C | 3:17078802 | TC | *TT* | TC | TT | *TC* | *TC* |  | TT | *TT* | *TT* |
| 76 |  | g.4047C>G | 3:17078830 | *CC* | *CC* | *CC* | CC | *CG* | CC |  | CC | *CC* | *CC* |
| 77 |  | g.4126C>A | 3:17078909 | *CC* | *CC* | *CC* | CC | *CA* | CC |  | CC | *CC* | *CC* |
| 78 |  | g.4133T>C | 3:17078916 | TC | TC | TC | *TC* | CC | *TC* |  | TT | *TT* | *TT* |
| 79 |  | g.4144C>T | 3:17078958 | *CC* | *CC* | CT | CC | *CT* | *CT* |  | CC | *CC* | *CC* |
| 80 |  | g.4843-4844del/ins 2 | 3:17079626-17079627 | del/ins | del/ins | del/ins | ins/ins | del/del | *del/ins* |  | ins/ins | *ins/ins* | *ins/ins* |
| 81 |  | g.4852A>G | 3:17079635 | AG | AG | AG | AA | *AG* | AA |  | AA | *AA* | *AA* |
| 82 |  | g.5067T>C | gap sequence | TC | TC | TC | TT | *TC* | *TC* |  | TT | *TT* | *TT* |
| 83 |  | g.5094G>A | gap sequence | GA | GA | *GG* | GG | GG | GG |  | GG | *GG* | *GG* |
| 84 |  | g.5174-5175del/ins 6 | gap sequence | *del/del* | *del/del* | del/ins | del/del | *del/ins* | *del/ins* |  | del/del | *del/del* | *del/del* |
| 85 |  | g.5267A>G | gap sequence | AG | AG | — | *AG* | — | — |  | AA | *AA* | *AA* |
| 86 |  | g.5381C>T | gap sequence | — | *CC* | — | *CT* | — | — |  | CC | *CC* | *CC* |
| 87 |  | g.5386C>T | gap sequence | — | CT | — | *CT* | — | — |  | CC | *CC* | *CC* |
| 88 |  | g.6060-6061del/ins 1 | gap sequence | del/ins | del/ins | — | *del/ins* | *del/ins* | *del/ins* |  | del/del | *del/del* | *del/del* |
| 89 |  | g.6150G>A | gap sequence | *GG* | *GG* | GA | GG | AA | GG |  | GG | *GG* | *GG* |
| 90 |  | g.6168T>C | gap sequence | *TT* | *TT* | TC | TT | CC | TT |  | TT | *TT* | *TT* |
| 91 |  | g.6206A>G | gap sequence | *AA* | *AA* | AG | AA | GG | AA |  | AA | *AA* | *AA* |
| 92 |  | g.6294T>C | gap sequence | *TT* | *TT* | TC | TT | CC | TT |  | TT | *TT* | *TT* |
| 93 | Intron5 | g.6539A>G | 3:17080373 | *AA* | *AA* | *AA* | *AG* | AA | AA |  | AA | *AA* | *AA* |
| 94 |  | g.6621C>T | 3:17080455 | *CC* | *CC* | *CC* | *CT* | CC | CC |  | CC | *CC* | *CC* |
| 95 |  | g.6623A>G | 3:17080457 | *AA* | *AA* | AG | *AG* | GG | AA |  | AA | *AA* | *AA* |
| 96 |  | g.6734C>T | 3:17080568 | *CC* | *CC* | *CC* | *CT* | *CT* | CC |  | CC | *CC* | *CC* |
| 97 |  | g.6801G>T | 3:17080635 | *GG* | *GG* | *GG* | *GT* | GG | GG |  | GG | *GG* | *GG* |
| 98 |  | g.6816T>C | 3:17080650 | *TT* | *TT* | *TT* | *TC* | *TC* | TT |  | TT | *TT* | *TT* |
| 99 |  | g.6881C>T | 3:17080715 | *CC* | *CC* | *CC* | *CT* | CC | CC |  | CC | *CC* | *CC* |
| 100 |  | g.6882A>G | 3:17080716 | *AA* | *AA* | *AA* | *AG* | AA | AA |  | AA | *AA* | *AA* |
| 101 |  | g.6896A>G | 3:17080729 | — | — | — | *AG* | — | — |  | AA | *AA* | *AA* |
| 102 | Exon6 | g.7044G>A | 3:17080878 | — | *GG* | GA | — | AA | — |  | GG | *GG* | *GG* |
| 103 | Intron6 | g.7104G>A | 3:17080938 | GA | *GG* | GA | — | AA | — |  | GG | *GG* | *GG* |
| 104 |  | g.7137T>C | 3:17080971 | TC | TC | TC | — | CC | — |  | TT | *TT* | *TT* |
| 105 |  | g.7181A>C | 3:17081014 | AC | AC | AC | — | CC | — |  | AA | *AA* | *AA* |
| 106 |  | g.7256C>G | 3:17081090 | *CC* | CG | *CC* | — | CC | — |  | CC | *CC* | *CC* |
| 107 |  | g.7316C>T | 3:17081149 | *CC* | CT | *CC* | — | CC | — |  | CC | *CC* | *CC* |
| 108 |  | g.7345A>T | 3:17081179 | AT | AT | AT | — | TT | — |  | AA | *AA* | *AA* |
| 109 |  | g.7406G>C | 3:17081239 | *GG* | *GG* | *GG* | — | GG | — |  | GG | *GG* | *GG* |
| 110 |  | g.7407T>C | 3:17081240 | *TT* | *TT* | *TT* | — | TT | — |  | TT | *TT* | *TT* |
| 111 | Exon7 | g.7495C>T | 3:17081328 | *CC* | CT | *CC* | — | CC | — |  | CC | *CC* | *CC* |
| 112 | Intron7 | g.7555C>G | 3:17081388 | *CC* | CG | *CC* | — | CC | — |  | CC | *CC* | *CC* |
| 113 |  | g.7594A>G | 3:17081428 | AG | *AA* | AG | — | GG | — |  | AA | *AA* | *AA* |
| 114 |  | g.7644G>C | 3:17081478 | GC | GC | GC | — | CC | — |  | GG | *GG* | *GG* |
| 115 | Intron8 | g.7893T>C | 3:17081726 | TC | *TT* | TC | TT | *TC* | TT |  | TT | *TT* | *TT* |
| 116 |  | g.7919T>A | 3:17081752 | TA | *TT* | TA | — | *TA* | TT |  | TT | *TT* | *TT* |
| 117 | Exon9 | g.7936T>C | 3:17081770 | *TT* | TC | *TT* | *TC* | *TC* | *TC* |  | TT | *TT* | *TT* |
| 118 | Intron9 | g.8118A>CC | 3:17081951 | *AA* | *AA* | A/CC | AA | *A/CC* | AA |  | AA | *AA* | *AA* |
| 119 |  | g.8162G>C or G>T | 3:17081996 | GC | GT | GT | *GC* | *CT* | *GT* |  | GG | *GG* | *GG* |
| 120 |  | g.8182T>C | 3:17082015 | TC | TC | TC | *TC* | CC | *TC* |  | TT | *TT* | *TT* |
| 121 |  | g.8184C>T | 3:17082017 | *CC* | *CC* | — | CC | — | *CT* |  | CC | *CC* | *CC* |
| 122 |  | g.8198A>C | 3:17082032 | AC | AC | AC | *AC* | CC | *AC* |  | AA | *AA* | *AA* |
| 123 |  | g.8252G>C | 3:17082086 | GC | *GG* | *GG* | GG | GG | GG |  | GG | *GG* | *GG* |
| 124 |  | g.8254-8255del/ins 1 | 3:17082088-17082089 | del/ins | del/ins | del/ins | *del/ins* | ins/ins | *del/ins* |  | del/del | *del/del* | *del/del* |
| **125** |  | **g.8283A>C** | **3:17082117** | **AC** | **AC** | **AC** | **CC** | **CC** | **CC** |  | **AA** | **AC** | **AC** |
| 126 | Exon10 | g.8314T>C | 3:17082148 | — | TC | TC | *TC* | CC | — |  | TT | *TT* | *TT* |
| 127 |  | g.8365C>T | 3:17082199 | *CC* | *CC* | *CC* | *CT* | CC | CC |  | CC | *CC* | *CC* |
| 128 |  | g.8602G>A | 3:17082436 | *GG* | *GG* | *GG* | *GA* | GG | GG |  | GG | *GG* | *GG* |
| 129 |  | g.8611G>A | 3:17082444 | GA | *GG* | *GG* | GG | GG | GG |  | GG | *GG* | *GG* |
| 130 |  | g.8648C>T | 3:17082482 | *CC* | *CC* | *CC* | *CT* | CC | CC |  | CC | *CC* | *CC* |
| 131 |  | g.8696T>C | 3:17082530 | *CC* | *TT* | TC | *TC* | CC | *TC* |  | TT | *TT* | *TT* |
| 132 |  | g.8730C>G | 3:17082564 | *CC* | *CC* | *CC* | *CG* | *CG* | CC |  | CC | *CC* | *CC* |
| 133 |  | g.8740-8741del/ins 11 | 3:17082574-17082575 | del/ins | *del/del* | *del/del* | del/del | del/del | del/del |  | del/del | *del/del* | *del/del* |
| 134 |  | g.8741C>T | 3:17082575 | *CC* | *CC* | *CC* | *CT* | *CT* | CC |  | CC | *CC* | *CC* |
| 135 |  | g.8745A>G | 3:17082579 | *AA* | *AA* | *AA* | AA | *AG* | AA |  | AA | *AA* | *AA* |
| 136 |  | g.8774T>C | 3:17082608 | *TT* | *TT* | *TT* | *TC* | *TC* | TT |  | TT | *TT* | *TT* |
| 137 |  | g.8789C>T | 3:17082622 | *CC* | *CC* | CT | CC | *CT* | *CT* |  | CC | *CC* | *CC* |
| 138 |  | g.8846G>A | 3:17082679 | *GG* | *GG* | GA | GG | *GA* | *GA* |  | GG | *GG* | *GG* |
| 139 |  | g.8873A>T | 3:17082707 | *AA* | *AA* | *AA* | *AT* | *AT* | AA |  | AA | *AA* | *AA* |
| 140 |  | g.8874G>A | 3:17082708 | *GG* | *GG* | GA | GG | *GA* | *GA* |  | GG | *GG* | *GG* |
| 141 |  | g.8916C>T | 3:17082750 | *CC* | *CC* | *CC* | *CT* | *CT* | CC |  | CC | *CC* | *CC* |
| 142 |  | g.8931T>C | 3:17082765 | *TT* | *TT* | *TT* | *TC* | *TC* | TT |  | TT | *TT* | *TT* |

^a^ The QTL genotypes of the 9 boars have been indicated in Table S3. The SNP genotypes inconsistent with the corresponding QTL genotypes are italicized. The locations of *PHKG1* genomic DNA mutations on pig draft genome sequences (Sscrofa10.2) are shown here. —, not genotyped.
