# Supplementary material for: A Splice Mutation in the PHKG1 Gene Causes High Glycogen Content and Low Meat Quality in Pig Skeletal Muscle
Source: PLoS Genet. 2014 Oct 23;10(10):e1004710. doi: 10.1371/journal.pgen.1004710 (PMC4207639; doi:10.1371/journal.pgen.1004710)
Supplement: Table S5 — Effect of PHKG1 g.8283C>A on meat quality traits of longissimus muscle and semimembranosus muscle in Chinese synthetic Sutai pigs. (DOCX) [file pgen.1004710.s014.docx]

**Table S5.** Effect of *PHKG1* g.8283C>A on meat quality traits of longissimus muscle and semimembranosus muscle in Chinese synthetic Sutai pigs

|  | Mean ± standard error ^b^ | | |  |
| --- | --- | --- | --- | --- |
| **Traits^a^** | **AA (n)** | **AC (n)** | **CC (n)** | ***P* value** |
| 24-h drip loss of SM, %, | 1.71±1.40^a^  (109) | 1.22±1.31^b^  (176) | 1.01±1.06^b^  (90) | 7.52E-07 |
| pH45min of LM | 6.53±0.39^a^  (92) | 6.45±0.46^a^  (162) | 6.31±0.42^b^  (93) | 0.141 |
| pH45min of SM | 6.65±0.40^a^  (92) | 6.54±0.48^a^  (162) | 6.4±0.41^b^  (93) | 0.039 |
| pH24h of SM | 5.55±0.14^a^  (99) | 5.66±0.22^b^  (158) | 5.77±0.23^c^  (92) | 4.58E-16 |
| pH drop from 45min to 24h in LM | 0.94±0.34^a^  (85) | 0.75±0.36^b^  (134) | 0.6±0.43^c^  (89) | 3.79E-06 |
| pH drop from 45min to 24h in SM | 1.06±0.37^a^  (88) | 0.77±0.42^b^  (139) | 0.6±0.42^c^  (89) | 6.94E-11 |
| Minolta L* (lightness) of LM | 47.79±2.70^a^  (122) | 48.46±4.08^a^  (203) | 48.16±3.95^a^  (104) | 5.71E-03 |
| Minolta L* (lightness) of SM | 44.02±2.64^a^  (122) | 44.68±3.88^a^  (203) | 44.01±3.39^a^  (104) | 2.00E-04 |
| Minolta a* (redness) of LM | 1.04±1.69^a^  (122) | 0.76±1.43^a^  (203) | 0.6±1.33^a^  (104) | 2.58E-02 |
| Minolta a* (redness) of SM | 4.28±1.78^a^  (122) | 3.22±1.58^b^  (203) | 3.13±1.79^b^  (104) | 1.66E-07 |
| Minolta b* (yellowness) of LM | 6.63±1.57^a^  (122) | 6.31±1.78^a^  (203) | 5.45±1.76^b^  (104) | 1.61E-03 |
| Minolta b* (yellowness) of SM | 7.40±1.91^a^  (122) | 6.58±1.96^b^  (203) | 5.63±2.18^c^  (104) | 4.92E-04 |
| Subjective color score of LM, 1-6 | 2.50±0.59^a^  (122) | 2.54±0.60^a^  (203) | 2.65±0.64^a^  (104) | 0.014 |
| Subjective color score of SM, 1-6 | 3.41±0.56^a^  (122) | 3.26±0.67^a^  (203) | 3.42±0.58^a^  (104) | 0.014 |
| Subjective marbling score of SM, 1-10 | 1.8±0.34^a^  (122) | 1.99±0.41^b^  (203) | 1.98±0.38^b^  (104) | 4.77E-04 |

^a^ LM, longissimus muscle; SM, semimembranosus muscle; pH45min, pH24h and pH36h, pH values measured at postmortem 45 min, 24 h and 36 h respectively.

^b^ Values with different superscripts in a row are significantly different from each other (*P* < 0.05).
