# Supplementary material for: A Splice Mutation in the PHKG1 Gene Causes High Glycogen Content and Low Meat Quality in Pig Skeletal Muscle
Source: PLoS Genet. 2014 Oct 23;10(10):e1004710. doi: 10.1371/journal.pgen.1004710 (PMC4207639; doi:10.1371/journal.pgen.1004710)
Supplement: Table S6 — Effect of PHKG1 g.8283C>A on carcass weight in White Duroc × Erhualian F2 intercross, Sutai pigs and Duroc × (Landrace × Yorkshire) hybrid pigs. (DOCX) [file pgen.1004710.s015.docx]

**Table S6.** Effect of *PHKG1* g.8283C>A on carcass weight in White Duroc × Erhualian F_2_ intercross, Sutai pigs and Duroc × (Landrace × Yorkshire) hybrid pigs.

|  | Mean ± standard error | | |  |
| --- | --- | --- | --- | --- |
| **Populations** | **AA (n)** | **AC (n)** | **CC (n)** | ***P* value** |
| F_2_ intercross | 71.44±16.03  (49) | 67.88±12.76  (367) | 65.83±13.85  (514) | 0.126 |
| Sutai | 46.97±11.69  (122) | 52.02±13.58  (205) | 55.50±10.20  (105) | 0.217 |
| DLY | 85.122±10.493  (9) | 82.893±7.908  (45) | 84.709±7.804  (85) | 0.757 |
