# Supplementary material for: A Splice Mutation in the PHKG1 Gene Causes High Glycogen Content and Low Meat Quality in Pig Skeletal Muscle
Source: PLoS Genet. 2014 Oct 23;10(10):e1004710. doi: 10.1371/journal.pgen.1004710 (PMC4207639; doi:10.1371/journal.pgen.1004710)
Supplement: Table S7 — The 53 SNPs detected by OpenArray genotyping platform. (DOCX) [file pgen.1004710.s016.docx]

**Table S7.** The 53 SNPs detected by OpenArray genotyping platform

| **No.** | **SNP name** | **Position (bp) on Chromosome 3^a^** | **Mutation** |
| --- | --- | --- | --- |
| 1 | AHY957S | 16862405 | C>T |
| 2 | AH0I4D0 | 16895991 | C>T |
| 3 | AH200QG | 16920601 | A>G |
| 4 | AH39YWO | 16936505 | C>T |
| 5 | AH5IW2W | 16951039 | G>A |
| 6 | AH6RU84 | 16976714 | C>T |
| 7 | AH70TFC | 17005809 | A>G |
| 8 | AHAA6RD | 17020712 | A>G |
| 9 | AHBJ4XL | 17031073 | T>C |
| 10 | AHCS23T | 17051047 | C>T |
| 11 | AHD1091 | 17062301 | G>A |
| 12 | AHFAZF9 | 17078916 | T>C |
| 13 | AHHSVSP | 17091063 | A>G |
| 14 | AHI1TYX | 17092585 | A>G |
| 15 | AHKAR45 | 17112826 | G>A |
| 16 | AHLJQBD | 17134159 | T>C |
| 17 | AHMSOHL | 17147009 | C>A |
| 18 | AHN1MNT | 17288109 | A>G |
| 19 | AHPAKT1 | 17304160 | T>C |
| 20 | AHQJIZ9 | 17320996 | C>T |
| 21 | AHRSG6H | 17244043 | C>T |
| 22 | AHUADIX | 17260371 | T>C |
| 23 | AHVJBO5 | 17159022 | G>A |
| 24 | AHWR9VD | 17231433 | G>A |
| 25 | AHX071L | 17218091 | A>G |
| 26 | AHY957T | 17339282 | A>G |
| 27 | AH0I4D1 | 17350789 | T>C |
| 28 | AH1R2J9 | 17367182 | G>C |
| 29 | AH39YWP | 17422132 | C>T |
| 30 | AH6RU85 | 17431311 | A>C |
| 31 | AH70TFD | 17385225 | C>T |
| 32 | AH89RLL | 17634573 | A>G |
| 33 | AHAA6RE | 17629424 | A>G |
| 34 | AHBJ4XM | 17605447 | G>A |
| 35 | AHCS23U | 17585740 | T>C |
| 36 | AHD1092 | 17566723 | T>C |
| 37 | AHFAZGA | 17555307 | G>C |
| 38 | AHGJXMI | 17536037 | C>T |
| 39 | AHHSVSQ | 17515976 | T>C |
| 40 | AHI1TYY | 17510471 | A>G |
| 41 | AHLJQBE | 17491579 | C>T |
| 42 | AHMSOHM | 17646321 | T>C |
| 43 | AHN1MNU | 17657261 | C>G |
| 44 | AHPAKT2 | 17668241 | T>C |
| 45 | AHQJI0A | 17729967 | A>G |
| 46 | AHRSG6I | 17744198 | A>G |
| 47 | AHS1FCQ | 17756943 | G>A |
| 48 | AHVJBO6 | 17982229 | C>T |
| 49 | AHWR9VE | 17958888 | T>C |
| 50 | AHX071M | 17948672 | A>C |
| 51 | AHY957U | 17859818 | T>C |
| 52 | AH0I4D2 | 17847552 | T>G |
| 53 | AH1R2KA | 17841591 | A>G |

^a^ The location of the mutation based on Sus scrofa genome assembly 10.2 (Sscrofa10.2)
