# Supplementary material for: A Splice Mutation in the PHKG1 Gene Causes High Glycogen Content and Low Meat Quality in Pig Skeletal Muscle
Source: PLoS Genet. 2014 Oct 23;10(10):e1004710. doi: 10.1371/journal.pgen.1004710 (PMC4207639; doi:10.1371/journal.pgen.1004710)
Supplement: Table S8 — Primers used for cDNA sequencing of the PHKG1 gene. (DOCX) [file pgen.1004710.s017.docx]

**Table S8.** Primers used for cDNA sequencing of the *PHKG1* gene^a^

| No. | Region | Forward primer name | Forward primer sequence (5’- 3’) | Reverse primer Name | Reverse primer sequence (5’- 3’) | Size (bp) |
| --- | --- | --- | --- | --- | --- | --- |
| 1 | Exon1-6 | 5’RACE Outer Primer | CATGGCTACATGCTGACAGCCTA | PHKG1-5' OUT | ATGCACGATGTCGAGCTTGTG | 600 |
| 2 | Exon1-6 | 5’RACE Inner Primer | CGCGGATCCACAGCCTACTGATGATCAGTCGATG | PHKG1-5' INNER | CATGATCTTCCTGGTTTCCTT | 549 |
| 3 | Exon10 | 3’RACE Outer Primer | TACCGTCGTTCCACTAGTGATTT | PHKG1-3' OUT | ACGCATTCCGAATCTATGGC | 600 |
| 4 | Exon10 | 3’RACE Inner Primer | CGCGGATCCTCCACTAGTGATTTCACTATAGG | PHKG1-3' INNER | GAAGGGGCAGCAGCAGAACC | 569 |
| 5 | Exon 1-4 | 5'RACE-1-FP | TATTCTGGCCACAAGTGTCTG | 5'RACE-1-RP | AAGAAGAAAGTGTTGGTCTCATAA | 458 |
| 6 | Exon 2-6 | 5'RACE-2-FP | CTCAGCACAGGGCTTCTAT | 5'RACE-2-RP | TCTTCCTGGTTTCCTTCTCA | 356 |
| 7 | Exon 5-8 | PHKG1-1-FP | AGAGGGGAGCTCTTTGAT | PHKG1-1 RP | CGGTGTCCGAGTAATCAT | 457 |
| 8 | Exon 8-10 | PHKG1-2-FP | AATGGGATGATTACTCGG | PHKG1-2 RP | TCAGTAGTCCTCGTCCTCA | 410 |
| 9 | Exon 9-10 | PHKG1-3-FP | CTGTTCGGCAGAAGAGGC | PHKG1-3-RP | CCCGGCGGTACTGGTAAT | 146 |
| 10 | Exon10 | 3'RACE-1-FP | GCTGAGGACGAGGACTACTG | 3'RACE-1-RP | GCTTCCCTTTCTATTGTTACTG | 494 |

^a^ The 5’RACE Outer Primer and 5’RACE inner Primer provided by 5’-Full Race Kit (Takara) are used for the first and the second step of a nested PCR reaction, respectively. The 3’RACE Outer Primer and 3’RACE inner Primer provided by 3’-Full Race Kit (Takara) are also used for the first and the second step of a nested PCR reaction, respectively.
