# Supplementary material for: A Splice Mutation in the PHKG1 Gene Causes High Glycogen Content and Low Meat Quality in Pig Skeletal Muscle
Source: PLoS Genet. 2014 Oct 23;10(10):e1004710. doi: 10.1371/journal.pgen.1004710 (PMC4207639; doi:10.1371/journal.pgen.1004710)
Supplement: Table S9 — Primers used for DNA sequencing of the PHKG1 gene. (DOCX) [file pgen.1004710.s018.docx]

**Table S9.** Primers used for DNA sequencing of the *PHKG1* gene.

| No. | Region | Forward primer sequence (5’- 3’) | Reverse primer sequence (5’- 3’) | Size (bp) |
| --- | --- | --- | --- | --- |
| 1 | Upstream 1 kb | GTGCTTTGTAAAATGCTAA | AGAGGGCTGAAGCGTGTC | 1065 |
| 2 | Exon1-Intron1 | GCCACAAGTGTCTGCTCGGG | AGCCAGGCGGGGAAAGTCTC | 618 |
| 3 | Intron1 | GAGACTTTCCCCGCCTGGCT | GGCTGGAGGTATCCCTGTGG | 670 |
| 4 | Intron1 | GGCATTCTGGGAACAGGACC | ACCTCCGAGAAACTCTGTGAAG | 600 |
| 5 | Intron1-Exon2 | CTTCACAGAGTTTCTCGGAGGT | GAAAGGCTCTAAGAAGGGAACAG | 673 |
| 6 | Exon2-Intron2 | CTTCTGTTCCCTTCTTAGAGCCT | AAGGTGTCCTGCCTTGTCCC | 583 |
| 7 | Intron2-Intron3 | CTCAAAGCTCACAGGAAGTTGG | GCTGTACTGAAATGGAAATGGC | 676 |
| 8 | Intron3-Intron4 | TAGCCATTTCCATTTCAGTACA | CTTCGGGTCCCTCCCTCCAC | 604 |
| 9 | Intron4 | GGAGGTGACCTGAGCAGACA | CACTAATAGCAGCAGCAGCAA | 775 |
| 10 | Intron4-Exon5 | TTTGCTGCTGCTGCTATTAGTG | TCACCTGGTTTCCTTCTCACTC | 1531 |
| 11 | Exon5-Exon6 | GTGAGAAGGAAACCAGGTGA | TGTTGTCGTCCAAGAGGATGTT | 551 |
| 12 | Exon6-Exon8 | GAACATCCTCTTGGACGACA | GTACATGATGACCCCTGTGC | 724 |
| 13 | Intron7-Exon10 | GCAGGTGGAGCACAGGGGTCA | GGGCGTAGGGGTCTCGGATG | 712 |
| 14 | Exon10 | GTGCTGGCTTCT GTGAGGAT | TCTATTGTTACTGATGGGAGGG | 698 |
