# Supplementary material for: A Splice Mutation in the PHKG1 Gene Causes High Glycogen Content and Low Meat Quality in Pig Skeletal Muscle
Source: PLoS Genet. 2014 Oct 23;10(10):e1004710. doi: 10.1371/journal.pgen.1004710 (PMC4207639; doi:10.1371/journal.pgen.1004710)
Supplement: Table S10 — Primers used for quantitative RT-PCR. (DOCX) [file pgen.1004710.s019.docx]

**Table S10.** Primers used for quantitative RT-PCR

| No. | Primer name | Sequence (5’-3’) | Size (bp) | Application |
| --- | --- | --- | --- | --- |
| 1 | Common-5'-FP | TGGGATGATTACTCGGACAC | 125 | Simultaneously amplifying wide-type transcript (Wt or q) and mutant-type transcript (Mt or Q) |
|  | Common -5'-RP | TCCTCCACCACGTACTGC |  |  |
| 2 | Wt-3'-FP | CTGTTCGGCAGAAGAGGC | 119 | Amplification of Wt only. |
|  | Wt-3'-RP | ACAGAAGCCAGCACCGTC |  |  |
| 3 | ACTB-FP | TCGCGGACAGGATGCAGAAAGA | 149 | Amplification ACTB house-keeping gene as a control |
|  | ACTB-RP | GCTGATCCACATCTGCTGGAA |  |  |
